# Multi-Omics Analysis of Circulating Exosomes in Adherent Long-Term Treated OSA Patients

**DOI:** 10.3390/ijms242216074

**Published:** 2023-11-08

**Authors:** Abdelnaby Khalyfa, Jose M. Marin, David Sanz-Rubio, Zhen Lyu, Trupti Joshi, David Gozal

**Affiliations:** 1 Department of Child Health, Child Health Research Institute, School of Medicine, University of Missouri, Columbia, MO 65211, USA; dgozal@missouri.edu; 2 Translational Research Unit, Hospital Universitario Miguel Servet & IISAragon, CIBERES, 50009 Zaragoza, Spain; 3 Department of Electrical Engineering and Computer Science, University of Missouri, Columbia, MO 65201, USA; zl7w2@mail.missouri.edu (Z.L.); joshitr@health.missouri.edu (T.J.); 4 Department of Health Management and Informatics, MU Institute for Data Science and Informatics and Christopher S Bond Life Science Center, University of Missouri, Columbia, MO 65211, USA; 5 Joan C. Edwards School of Medicine, Marshall University, Huntington, WV 25755, USA

**Keywords:** OSA, exosomes, extracellular vesicles, lipids, proteomics, miRNAs, omics, multi-omics

## Abstract

Obstructive sleep apnea (OSA) is a highly prevalent chronic disease affecting nearly a billion people globally and increasing the risk of multi-organ morbidity and overall mortality. However, the mechanisms underlying such adverse outcomes remain incompletely delineated. Extracellular vesicles (exosomes) are secreted by most cells, are involved in both proximal and long-distance intercellular communication, and contribute toward homeostasis under physiological conditions. A multi-omics integrative assessment of plasma-derived exosomes from adult OSA patients prior to and after 1-year adherent CPAP treatment is lacking. We conducted multi-omic integrative assessments of plasma-derived exosomes from adult OSA patients prior to and following 1-year adherent CPAP treatment to identify potential specific disease candidates. Fasting morning plasma exosomes isolated from 12 adult patients with polysomnographically-diagnosed OSA were analyzed before and after 12 months of adherent CPAP therapy (mean ≥ 6 h/night) (OSAT). Exosomes were characterized by flow cytometry, transmission electron microscopy, and nanoparticle tracking analysis. Endothelial cell barrier integrity, wound healing, and tube formation were also performed. Multi-omics analysis for exosome cargos was integrated. Exosomes derived from OSAT improved endothelial permeability and dysfunction as well as significant improvement in tube formation compared with OSA. Multi-omic approaches for OSA circulating exosomes included lipidomic, proteomic, and small RNA (miRNAs) assessments. We found 30 differentially expressed proteins (DEPs), 72 lipids (DELs), and 13 miRNAs (DEMs). We found that the cholesterol metabolism (has04979) pathway is associated with lipid classes in OSA patients. Among the 12 subjects of OSA and OSAT, seven subjects had complete comprehensive exosome cargo information including lipids, proteins, and miRNAs. Multi-omic approaches identify potential signature biomarkers in plasma exosomes that are responsive to adherent OSA treatment. These differentially expressed molecules may also play a mechanistic role in OSA-induced morbidities and their reversibility. Our data suggest that a multi-omic integrative approach might be useful in understanding how exosomes function, their origin, and their potential clinical relevance, all of which merit future exploration in the context of relevant phenotypic variance. Developing an integrated molecular classification should lead to improved diagnostic classification, risk stratification, and patient management of OSA by assigning molecular disease-specific therapies.

## 1. Introduction

Obstructive sleep apnea (OSA) is a worldwide public health problem affecting nearly one billion people [1,2,3]. OSA is characterized by recurring upper airway obstructive events during sleep that are contingent on their characteristics and duration, and result in increased respiratory efforts, intermittent hypoxemia (IH), periods of elevated carbon dioxide levels in blood, autonomic surges, and episodic arousals leading to sleep fragmentation (SF) [4,5,6,7]. Such events then lead to activation of pathophysiological pathways that are complex and multifactorial, with many yet unrecognized and poorly understood facets. Indeed, OSA is now identified as an independent risk factor for cardiovascular morbidities encompassing conditions such as systemic and pulmonary hypertension, arrhythmias, coronary heart disease, stroke, heart failure, and cancer, as well as metabolic diseases such as dyslipidemia and diabetes mellitus, in addition to neurocognitive impairments and depression, collectively resulting in reduced quality of life and enhanced overall mortality rates [6,8,9,10,11,12,13,14].

The high prevalence of undiagnosed and untreated OSA is also believed to be a major contributor to the morbidity and mortality of various highly prevalent cardiovascular, metabolic, and oncologic diseases [13,15,16,17]. The gold standard for diagnosing OSA is an overnight polysomnographic (PSG) test, from which multiple indices of severity are computed, with the Apnea-Hypopnea Index (AHI) currently serving as the most frequently used index to classify OSA severity (AHI > 5 events/h of sleep being considered abnormal and AHI > 30 events/h of sleep corresponding to severe OSA) [12]. Positive airway pressure (PAP) therapy is the preferred treatment for most people suffering from OSA, although a significant proportion of patients underuse it or reject it due to discomfort, insurance issues, and perceived inefficacy. Although this treatment improves OSA comorbidities [17,18,19,20,21], the mechanisms involved in such improved outcomes remain unknown. Endothelial dysfunction is an early ubiquitous event preceding many of the clinical manifestations of OSA [22]. Therefore, examining the molecular basis of OSA and its potential effects on endothelial function can improve our understanding of the mechanistic underpinnings of OSA-related adverse consequences.

In OSA patients, plasma-derived extracellular vesicles can induce endothelial dysfunction, suggesting that circulating extracellular vesicles are important messengers linking OSA to end-organ dysfunction [23,24,25,26,27,28]. Extracellular vesicles (EVs) are heterogeneous nanoparticles secreted and released from cells of all tissue types and carry cell- and body-fluid-specific signatures [29,30]. Exosomes, one of the several EV sub-types, are critical mediators of intercellular communication and regulation, capable of influencing the transcriptional landscape of target cells through horizontal transmission of biological cargos including proteins, lipids, and RNA species [28,30,31]. Thus, the study of exosomes and their cargos could provide significant and valuable insights into the mechanisms behind cell–cell communication and disease development and progression in OSA. Many studies have focused on evaluation of exosome cargos using isolated transcriptomic, proteomic, metabolomic, and lipidomic approaches [32,33]. However, although analysis of plasma exosomes by multi-omic approaches may provide unparalleled insights into disease mechanisms aimed at personalized medicine [34], and even though such multi-omic approaches have been successfully applied in other contextual settings [35], we are unaware of any studies to date that have focused on exosomes in OSA.

Here, we implemented state-of-the-art multi-omic approaches on circulating exosomes obtained from patients with moderate to severe OSA before and following long-term PAP-adherent treatment to gain increased understanding of putative molecular mechanisms underlying the disease, as well as to investigate some of the effects on naïve endothelial cell functions [36,37,38].

## 2. Results

### 2.1. Subject Characteristics

A total of 12 adult male subjects were recruited in this study, completing the 12-month adherent CPAP treatment after being diagnosed with severe OSA, as shown in Figure S1. There were no significant differences in BMI before and after treatment. However, AHI was markedly higher in OSA (70.13 ± 16.77 events/h of sleep) compared to during CPAP treatment (OSAT: 3.44 ± 2.171/events/h; *p* = 0.001). We also found that triglycerides (TG), total cholesterol (TC), high-density lipoprotein (HDL), and glucose were significantly decreased in OSAT compared to OSA, for example, the value for TG in OSA (222.14 ± 74.18) vs. OSAT (148.15 ± 78.17), TC mg/dL (222.14 ± 74. 18 vs. 148.15 ± 78.17, *p* = 0.01), HDL mg/dL (234.07 ± 20.14 vs. 212.28 ± 33.32, *p* = 0.02), and glucose ng/mL (101.12 ± 11.16 vs. 94.09 ± 14.28, *p* = 0.03), as shown in Table 1. In addition, significant differences emerged in diastolic blood pressure (dBP) between OSA (82.31 ± 5.3 mmHg) and OSAT (73.6 ± 10.23 mmHg; *p* = 0.01). The SpO_2_ during wake state was statistically significantly lower in OSA (90.50 ± 2.87%) compared with OSAT (94.70 ± 1.60%; *p* = 0.03) Table 1.

### 2.2. Exosome Characterization and Cellular Internalization

Differential centrifugation followed by polymer-based precipitation and filtration were used to isolate exosome-enriched OSA plasma samples prior to and following adherent CPAP treatment for 1 year. Plasma exosome isolation, characterization, and quantification are shown in Figure S2. Exosome concentrations were determined by Nanoparticle Tracking Analysis (NTA), and exosomes from OSA and OSAT displayed similar size distributions, with average range of 9.91 × 10^9^ mL^−1^ in OSA, and 8.82 × 10^9^ mL^−1^ in OSAT, respectively (Figure S2a). Exosome size was confirmed by negative stain transmission electron microscopy; their morphology showed the typical cup-shaped feature and ranged from 30 to 150 nm in diameter (Figure S2b), confirming published results [39].

We further confirmed the presence of typical tetraspanin exosome markers (CD63 and CD81) using the ExoView R100 (System Biosciences, LLC, Palo Alto, CA, USA) platform and the proprietary antibody array (Figure S2c). Flow cytometry of isolated exosomes from OSA and OSAT groups revealed the presence of tetraspanin markers as anticipated from highly purified (>98%) exosome fractions (Figure S2c). Next, we compared the cellular internalization of autologous OSA- and OSAT-derived exosomes on naïve endothelial cells using PKH67 as a reporter (Figure S3). Endothelial uptake of exosomes from OSA and OSAT was similar for all subjects. The PKH67 signal was observed in the lipid cell membrane of cells grown in medium supplemented with PKH67-labelled exosomes, whereas no signal was observed in cells grown in medium supplemented without exosomes to which PKH67 was also added.

### 2.3. Endothelial Barrier Integrity and Wound Healing

**Figure 1:**
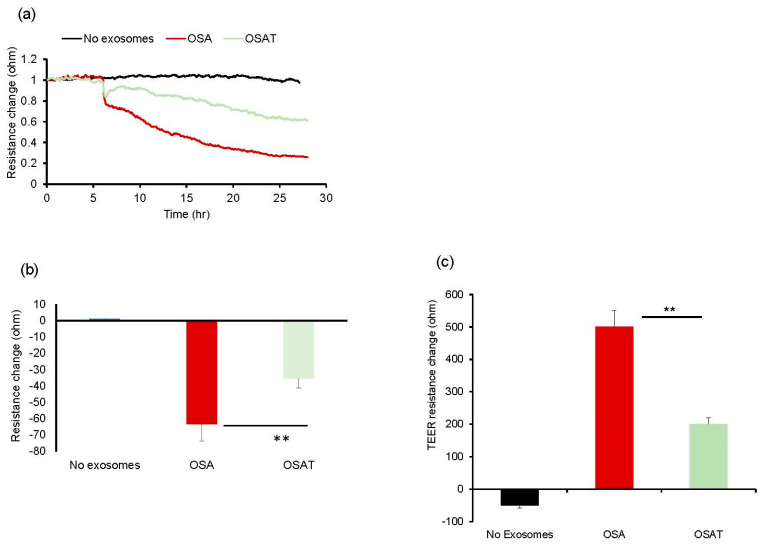
Exosomes derived from OSA subjects disrupt endothelial cell monolayer barrier integrity in vitro. (**a**) Ensemble-averaged curves of ECIS-measured endothelial cell barrier resistance changes over time after administration of exosomes from adult patients with OSA before treatment and after long-term-adherent CPAP (OSAT) therapy compared to endothelial cells incubated with plasma-free media and empty exosomes (control; black line). (**b**) Evaluation of ECIS-measured endothelial cell barrier resistance changes after exosome administration. Endothelial cells were grown on trans-well membranes to measure the integrity and permeability of the monolayer cells. (**c**) Continuous measurement of cell monolayer barrier function (TEER) using membrane inserts in multiple wells to measure the resistance across the trans-well membrane over time. TEER values for the average of each group: no exosomes, OSA, and OSAT. ** *p* < 0.001.

Next, we tested the impact of OSA and OSAT exosomes on human endothelial cell barrier integrity and changes in endothelial barrier impedance using ECIS. Exosomes from OSA patients induced greater disruption of the endothelial monolayer barrier integrity compared with OSAT (Figure 1a,b). The normalized resistance change was higher in OSA (−63 ± −6.81%) compared with OSAT (−35.83 ± −4.84%) (*p* = 0.001). We also used TEER to confirm the integrity and permeability of the endothelial cell monolayer. We found that the TEER values in trans-wells treated with exosomes derived from OSA were 6.1 ± 0.62 Ω·cm^−2^ compared with 3.73 ± 0.42 Ω·cm^−2^ for OSAT (*p* = 0.001). The TEER-normalized values were 500.67 ± 48.25 for OSA and 200.82 ± 20.48 for OSAT (*p*-value 0.003) (Figure 1c).

**Figure 2:**
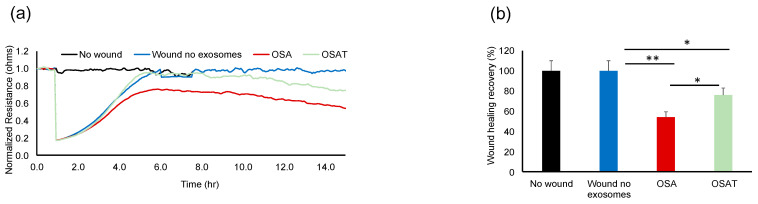
Plasma-exosome-induced wound healing in human endothelial cells. Comparative analysis of real-time endothelial barrier integrity following wounding on 8W10E arrays. Plasma-derived exosomes from OSA and OSAT patients applied to human microvascular endothelial cells (HMVEC-d) for wound healing using ECIS system in vitro. (**a**) Representative graphs of comparative analysis of hMVEC-d cells treated with and without exosomes following wound healing. HMVEC-d cells were seeded at 0 h at a density of 50,000 cells into ECIS system arrays (8W10E) for 24 h; cells were wounded, then exosomes derived from OSA (n = 12) and OSAT were added and monitored for another 48 h. (**b**) Histogram showing the effects of exosome cargos derived from OSA or OSAT, as well as unwounded cells and wounded cells with no exosomes, on endothelial cell wound healing and recovery of HMVEC-d cells. * Indicates *p* < 0.01, while ** *p* < 0.001.

The functional relevance of exosome transfer between HMVEC-d cells were investigated using ECIS in a wound-healing assay (Figure 2a). ECIS has also been used as an electric wound-healing assay to monitor cell migration [40]. Indeed, exosomes derived from OSA applied to HMVEC-d cells exhibited significantly slower recovery (54.23% ± 6.12%) when compared with OSAT (76.28% ± 8.21%; *p* = 0.001), as shown in Figure 2a,b, respectively. A quick drop in barrier resistance is observed when the dead cells detach from the electrode surface. Following this, cells migrate into the wounded area, and the impedance gradually increases and reflects the healing process. This automated assay has the advantage that the wound area is well defined and highly reproducible.

### 2.4. Angiogenesis (Tube Formation Assay)

**Figure 3:**
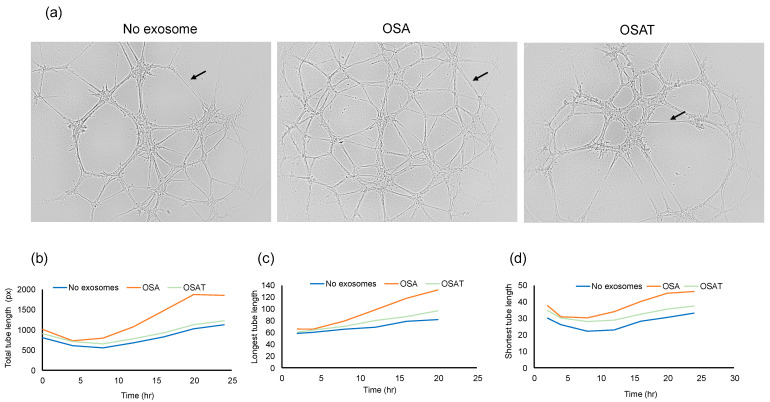
Plasma exosomes altered human endothelial cell tube formation (angiogenesis) in vitro. Plasma-derived exosomes from OSA or OSAT were applied on a 3-D matrix endothelial cell culture system to assess angiogenesis, and tube lengths were quantified using ImageJ software 2.9.0. The formation of tube-like structures was observed under bright field. (**a**) Representative of phase contrast micrographs of the capillary-like tubular structures of OSA and OSAT and compared with no exosomes for 24 h. Arrow indicates tube formation. (**b**) Line graphs showing total tube length, (**c**) longest tube length, and (**d**) shortest tube length. Tube formation was quantified by counting the number of branching points in the total photographed area. n = 12. Scale bar 100 μm.

To determine the functional effects of exosomes on endothelial cell function, we evaluated whether exosome internalization could induce endothelial tube formation. The cells were seeded at 30,000 cells/mL/well in 96-well plates coated with Matrigel. Brightfield images depicting tube formation are shown in Figure 3a. The tube formation was quantified using the number of tubes and length per tube (Figure 3b). No significant differences in the total area between endothelial cells treated with exosomes derived from OSA, OSAT, or cells not exposed to exosomes emerged (Figure 3b). The total tube length (OSA vs. OSAT, *p* = 0.004), longest tube (OSA vs. OSAT, *p* = 0.007), and shortest tube (OSA vs. OSAT, *p* = 0.001) were significantly higher in OSA-untreated cells compared with those treated with exosomes from OSAT subjects (Figure 3b,c). The largest tube formation was observed in OSA after 20 h compared with OSAT or cells not treated with exosomes. Overall, these results suggest that exosomes derived from OSA enhance migration and tube formation, and foster capillary-like structures, as opposed to OSAT.

### 2.5. Exosome Cargos

To study exosome cargos derived from OSA and OSAT, we performed comprehensive multi-omics analyses including delineation of their lipidomes using tandem mass spectroscopy (LC-MS/MS), miRNA content by next generation sequencing, and proteomics.

### 2.6. Lipidomic Analysis

**Figure 4:**
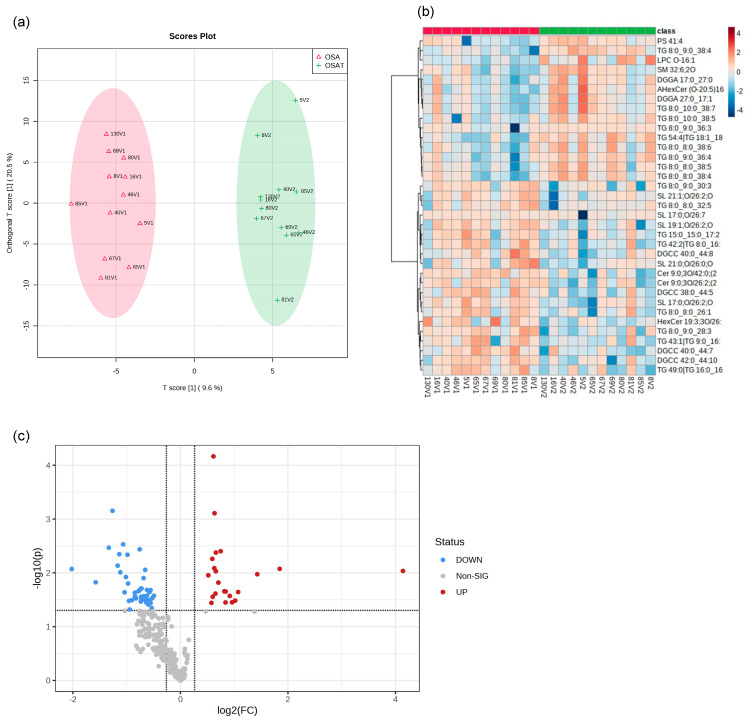
Lipidomic analysis of OSA exosomes using LC-MS/MS. (**a**) Supervised OPLS-DA model showing separation of OSA and OSAT groups. (**b**) Heatmap showing the top differentially expressed lipids. (**c**) Volcano plot depicting the total differentially expressed lipids. The red color indicates up-regulation, and the blue color indicates down-regulation. n = 12/group.

We determined exosome lipid composition profiles in 12 OSA subjects before and after 1-year adherent CPAP treatment. A total of 311 lipid compounds in OSA and OSAT plasma exosomes were identified, and these lipids were classified into four classes, i.e., sphingolipids (3.68% ± 0.14), glycerolipids (51.4% ± 1.15), phospholipids (38.41% ± 1.74), and saccharolipids (6.51% ± 0.92). We then studied the lipids characteristic of OSA compared to OSAT, based on the differential lipid abundance, using multivariate statistical analysis. Figure 4 shows the orthogonal partial least square discriminant analysis (OPLS-DA) for the separation of two groups, including total lipids (Figure 4a). The distributions of differentially abundant lipids are also displayed in heatmap form for total and subclass lipids (Figure 4b heatmap). Using volcano plots, we identified 72 lipid molecules that were statistically significant in total lipids (Figure 4c) with log_2_ fold changes and log_10_ *p*-value, respectively (fold change > 2 or <0.05 in Figure 4c and Table 2).

Furthermore, we show OPLS-DS, heatmap and volcano plot for lipids subclasses including sphingolipids, glycerolipids, phospholipids, and saccharolipids (Figures S4–S6), respectively. The data for OPLS-DS sphingolipids are shown in Figure S4a, for glycerolipids in Figure S4b, for phospholipids in Figure S4c, and for saccharolipids in Figure S4d. Heatmaps for lipid subclasses are shown in Figure S5: sphingolipids (Figure S5a), glycerolipids (Figure S5b), phospholipids (Figure S5c), and saccharolipids (Figure S5d). Using volcano plots, we identifiy differentially expressed lipids (log_2_) of sphingolipids (n = 5 lipids) in Figure S6a, glycerolipids (n = 39 lipids) in Figure S5b, phospholipids (n = 5 lipids) in Figure S6c, and saccharolipids (n = 2 lipids) in Figure S6d.

To investigate whether these lipids could be used as biomarkers for the diagnosis of OSA, we constructed a receiver operating characteristic (ROC) curve for the differential expressed lipids and investigated the area under the curve (AUC), which ranged between 0.95 and 0.70 (Supplementary Table S1). We found the highest ROC values were for TG 8.0_9.0_38.4 and Cer 9.0 30/42; Supplementary Table S1. The ROC for glycerolipids is shown in Supplementary Table S2, while the ROCs for phospholipids, saccharolipids, and sphingolipids are shown in Supplementary Table S3. The highest AUCs for individual glycerolipids were TG 8:0_9:0_38:4 (AUC = 0.94, *p* = 0.0002), and TG 54:4|TG 18:1_18:1_18:2 (AUC = 0.85, *p* = 0.002); for phospholipids: PS 41:4 (AUC = 0.83, *p* = 0.005), and LPC O-16:1 (AUC = 0.81, *p* = 0.008); for saccharolipids: SL 19:1;O/26:2;O (AUC = 0.85, *p* = 0.0008), SL 17:0;O/26:2;O (AUC = 0.82, *p* = 0.0007); and for sphingolipids: SM 32:6;2O (AUC = 0.82, *p* = 0.82), and Cer 9:0;3O/42:0;(2OH) (AUC = 0.81, *p* = 0.003). Thus, if confirmed in larger studies, these lipids may be used to identify a circulating plasma lipidomic signature that would allow the identification of subjects with OSA compared with OSAT or those with specific phenotypes [41].

To correlate lipids with metabolic function, we performed PatternHunter [42] using the differentially expressed lipids based on their component identification number (CID) and found that glycerolipid metabolism, metabolic pathways, thermogenesis, regulation of lipolysis in adipocytes, insulin resistance, fat digestion and absorption, vitamin digestion and absorption, cholesterol metabolism, and lipid and atherosclerosis were differentially affected following CPAP treatment.

### 2.7. Exosome Proteomic Analysis

**Figure 5:**
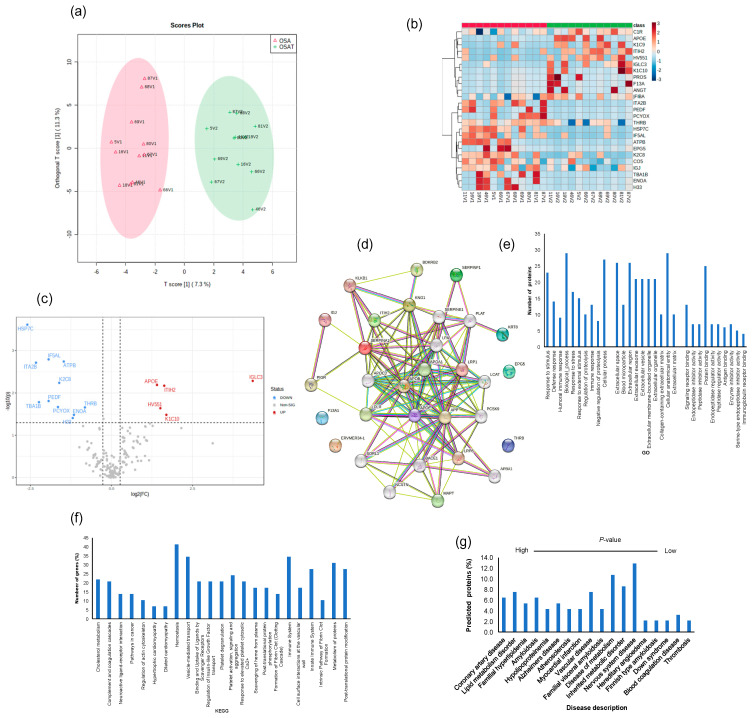
Proteomic analysis of OSA and OSAT using in situ digestion on LC-MS. (**a**) Orthogonal partial least square discriminant analysis (OPLS-DA) for the separation of two groups, (**b**) heatmap analysis, (**c**) volcano plots and (**d**) protein–protein network for differentially expressed proteins. The red color indicates up-regulation, and the blue color indicates down-regulation. n = 12/group. Temporal changes of gene ontology and KEGG pathways for exosome proteomics analysis for OSA and OSAT subjects. The differentially expressed proteins (DEPs) were subjected to KEGG, GO, and disease description. (**e**) Top terms from GO functional enrichment analyses based on DEPs of biological processes (BP), cellular components (CC) and molecular functions. (**f**) Top KEGG pathways based on pathway enrichment analysis KEGG) of DEPs pathways, and (**g**) disease description. All proteins associated with disease tissue networks were identified according to the DISEASES database.

To explore potential differences in the protein cargo among exosomes released into the plasma of OSA subjects before and after 1-year treatment, we subjected the isolated exosomes to liquid LC-MS/MS-based proteomics. In total, 190 proteins were identified, and 20 proteins showed statistically significant differences between OSA and OSAT samples. Accordingly, proteins with 1.2-fold change and *p* < 0.05 were determined as differentially expressed proteins (DEPs). To understand the potential functional impacts of proteins enriched in OSAT vs. OSA exosomes, we used OPLS-DA (Figure 5a), heatmap analysis (Figure 5b), and volcano plots (Figure 5c) of the differentially expressed proteins (DEPs) with log_2_ fold changed above 1.2 and log_10_-*p*-value as shown in Figure 5c). The data in volcano plots revealed that 16 highly significant proteins were detected and that 11 were down-regulated and 5 were up-regulated (Figure 5c). The down-regulated proteins and their log_2_ fold changes (FC) are HSP7C (FC = −2.59, *p* = 0.0002), ITA2B (FC = −2.32, *p* = 0.001), TBA1B (FC = −2.23, *p* = 0.016), IF5AL (FC = −1.93, *p* = 0.0016), PEDF (FC = −1.94, *p* = 0.015), PCYOX (FC = −1.65, *p* = 0.021), K2C8 (FC = −1.61, *p* = 0.005), ATPB (FC = −1.46, *p* = 0.001), H33 (FC = −1.19, *p* = 0.04), ENOA (FC = −1.16, *p* = 0.03), and THRB (FC = −0.82, *p* = 0.021). The up-regulated proteins and their log_2_ fold changes are APOE (FC = 1.41, *p* = 0.006), HV551 (FC = 1.51, *p* = 0.022), ITIH2 (FC = 1.63, *p* = 0.006), K1C10 (FC = 1.69, *p* = 0.032), and IGLC3 (FC = 4.36, *p* = 0.005).

The differentially expressed proteins were further analyzed using the STRING database for enrichment gene ontology (GO) and pathway analysis [43] to predict the protein–protein interaction (PPI) and to identify hub genes of the DEPs. The top highly significant pathways were cholesterol metabolism (*p* = 0.00001), complement and coagulation cascades (*p* =3.55 × 10^−19^), neuroactive ligand–receptor interaction (*p* = 0.001), pathways in cancer (*p* = 0.005), regulation of actin cytoskeleton (*p* = 0.007) hypertrophic cardiomyopathy (*p* = 0.004), dilated cardiomyopathy (*p* = 0.003), and hemostasis (*p* = 2.21 × 10^−4^), as shown in Figure 5d. We analyzed these proteins using GO enrichment analysis and selected the top 10 genes for biological process (PB), cellular component (CC), and functional process, as shown in Figure 5e. We found that exosome proteins are enriched in the extracellular region (45%), extracellular space (39%), organelle (39%), membrane (30%), cytoplasm (28%), extracellular exosome (27%), and cell surface (12%) (Figure 5e). In biological process, cellular processes (50%) and metabolic processes (40%) accounted for the majority, while for molecular functions, we found signaling receptor binding (9%), lipid binding (9%), and antigen binding (6%) as the leading processes (Figure 5e). To further understand the functions of the DEPs, KEGG analysis was performed. The significant KEGG pathway analyses are shown in Figure 5f. Here, we highlight some of the pathways with potential biological significance in OSA, such as cholesterol metabolism (*p* = 1.2 × 10^−5^), complement and coagulation cascades (*p* = 3.4 × 10^−19^), hemostasis (*p* = 1.7 × 10^−15^), and the immune system (*p* = 7.3 × 10^−15^) (Figure 5f).

Of the DEPs identified, several seem to be involved in known diseases, including coronary artery disease (PLAT, APOB, APOA1, APOE, PCSK9, LPA, FDR 0.00000001), lipid metabolism disorder (APOC3, APOB, APOA1, APOE, LCAT, PCSK9, LDLR, FDR 0.00000002), vascular disease (PLAT, SERPINE1, APOB, APOA1, APOE, PCSK9, LPA, FDR, 0.000007), atherosclerosis (APOB, APOA1, APOE, LPA, FDR, 0.000007), and neurodegenerative disease (APOE, SORL1, APP, BACE1, MAPT, TREM2, VLDLR, FDR, 0.0004) (Figure 5g). We also found DEPs involved in different organs and tissues, including bone marrow cells, digestive glands, plasma cells, and the skeletal system, liver, respiratory system, hematopoietic system, and cardiovascular system (Figure 5g).

As with proteomic differences, ROC curves and AUCs were constructed for 17 DEPs for OSA diagnosis, and their ROCs ranged from 0.90 to 0.60 (Figure S7). Of the 17 proteins, HSP7C had the highest AUC value (0.90, 95% confidence interval (CI): 0.767–0.98), with a sensitivity of 80% and specificity of 80%. The second-highest AUC value was for ATPB (0.85, 95% CI: 0.64–0.998), with a sensitivity of 100% and specificity of 70%, followed by IF5ALS (0.85, 95% CI: 0.64–0.99), with a sensitivity of 80% and specificity of 80.0%.

### 2.8. Exosomal miRNA Profile

**Figure 6:**
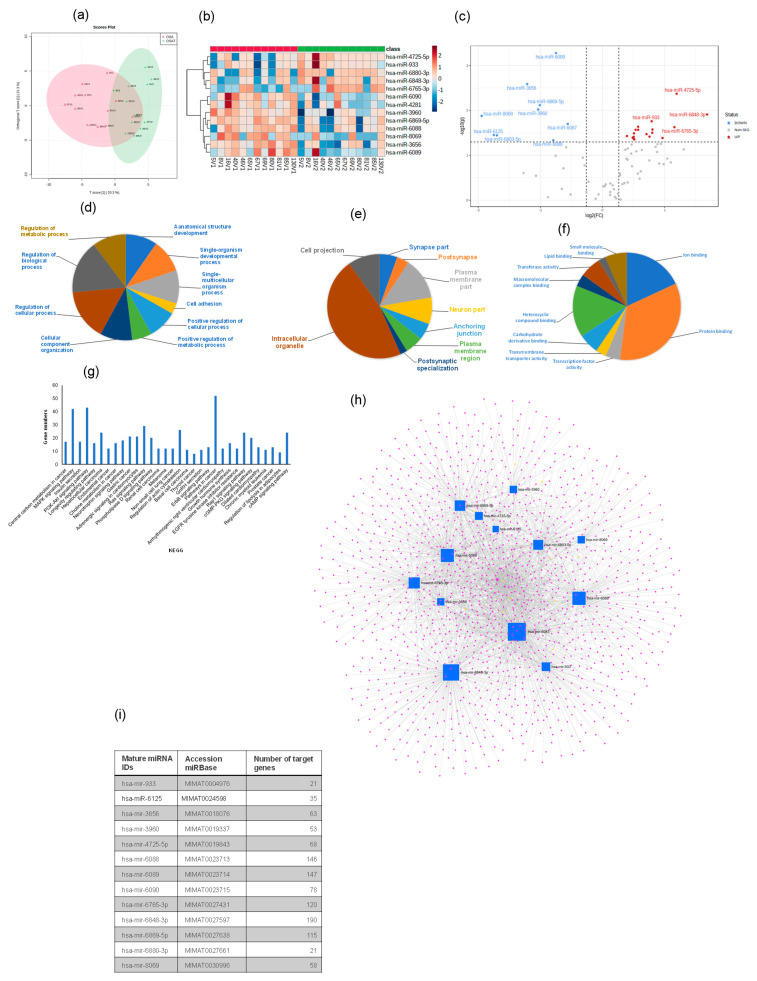
Plasma-derived exosome miRNA profiling for OSA and OSAT. (**a**) Orthogonal partial least square discriminant analysis (OPLS-DA); (**b**) Heatmap illustrating miRNA expression patterns in exosomes (dark red: increased miRNA expression; light blue: reduced miRNA expression). The dendrograms show hierarchical clustering representing the similarities and dissimilarities in expression profiles among individuals and miRNAs; and (**c**) volcano plots. Gene Ontology (GO) for the target prediction of differentially expressed miRNAs (miRNAs) in exosomes derived from OSA and OSAT subjects. GO analysis for (**d**) cellular components, (**e**) biological processes, and (**f**) molecular functions, and (**g**) KEGG pathways identified in target predication genes found in differentially expressed miRNAs in OSAT vs. OSA. Network visualization of the differentially expressed exosomal miRNAs derived from OSA and OSAT. (**h**) Network for 13 miRNAs, and (**i**) list of the miRNAs and their associated target predication genes. n = 12.

A total of 2529 human mature miRNAs were identified, and of these, 81 differentially expressed miRNAs were detected in all exosome samples. Heatmap analysis (OPLS-DA) revealed consistent and significant differences in miRNA expression profiles for exosomes from OSAT vs. OSA (Figure 6a,b). The score plot for OSA and OSAT using OPLS-DS plot is shown in Figure 6a, while the heatmap for the 13 most differentially expressed miRNAs is also shown (Figure 6b) and volcano plots (Figure 6c). Of those 13 miRNAs, there were 4 up-regulated and 9 down-regulated (Figure 6c). The up-regulated miRNAs were the following: hsa-miR-933 (fold = 0.79, *p* = 0.017), hsa-miR-6765-3p (fold = 1.16, *p* = 0.023), hsa-miR-4725-5p (fold = 1.19, *p* = 0.004), and hsa-miR-6848-3p (fold = −1.68, *p* = 0.012), while the down-regulated miRNAs were hsa-miR-8069 (fold = −1.95, *p* = 0.013), hsa-miR-6125 (fold = −1.75, *p* = 0.035), hsa-miR-6803-5p (fold = −1.70, *p* = 0.035), hsa-miR-3656 (fold = −1.21, *p* = 0.0025), hsa-miR-3960 (old = −1.037, *p* = 0.009), hsa-miR-6869-5p (fold = −1.09, *p* = 0.007), hsa-miR-6088 (fold = −0.79, *p* = 0.046), hsa-miR-6089 (fold = −0.74, *p* = 0.0005), and hsa-miR-6087 (fold = −0.56, *p* = 0.020). We used several computational databases for the target predictions of these 13 miRNAs and identified 2529 individual gene targets.

Next, GO of predicted mRNA targets were clustered to reveal the enriched molecular functions of target genes. Those genes were involved in many biological processes (BPs), including cell adhesion (*p* = 2.3 × 10^−14^), positive regulation of cellular processes (*p* = 2.3 × 10^−10^), positive regulation of metabolic processes (*p* = 6.4 × 10^−8^), and regulation of metabolic processes (*p* = 1.9 × 10^−6^) (Figure 6d–f), respectively. The miRNAs were also involved in many CC activities, including the intercellular organelle, plasma membrane, and cell projection (Figure 6f). The miRNAs were involved in many aspects of MFs, including lipid binding, heterocyclic compound binding, protein binding, and ion binding (Figure 6f). Canonical KEGG pathway analyses indicated that the differentially expressed miRNAs were involved in MAPK signaling (*p* = 3.03 × 10^−5^), insulin secretion (*p* = 3.7 × 10^−4^), the PI3K-Akt signaling pathway (*p* = 8.9 × 10^−4^), adrenergic signaling in cardiomyocytes (*p* = 0.005), choline metabolism in cancer (*p* = 0.004), neurotrophin signaling (*p* = 0.005), gastric cancer (*p* = 0.005), melanoma (*p* = 0.01) and non-small cell lung cancer (*p* = 0.01) (Figure 6g). To evaluate the probability of diagnosis with OSA, ROC curves were constructed and AUCs were calculated. The AUCs were: hsa-miR-6089 (0.87), hsa-miR-3960 (0.83), hsa-miR-3656 (0.81), and hsa-miR-6088 was (0.70) Figure S8. The AUC, *p*-value, and fold change for all miRNAs from 0.87–0.70 are presented in Figure S8.

We further validated four of the differentially expressed miRNAs (2 up-regulated and 2 down-regulated) using qRT-PCR. For up-regulated miRNAs, hsa-miR-6848 (1.72 ± 0.18-fold change (FC); *p* = 0.002), hsa-miR-4725 (1.21 ± 0.15 FC; *p* = 0.001) confirmed the findings, while down-regulated miRNAs were hsa-miR-6089 (−1.93 ± 0.19 FC; *p* = 0.003) and hsa-miR-3656 (−1.74 ± 0.19 FC; *p* = 0.01). Validated miRNAs showed similar expression differences to those identified in the array experiments. Next, we used miRNet [44], a web-based tool designed for creation, customization, visual exploration, and functional interpretation of miRNA–target interaction networks. The network for the 13 miRNAs and their associated genes is shown in Figure 6h, while the list of these miRNAs and their target prediction genes is shown in Figure 6i. This miRNet allows navigation of the complex landscape of miRNA–target interactions.

### 2.9. Multi-Omic Data Integration

**Figure 7:**
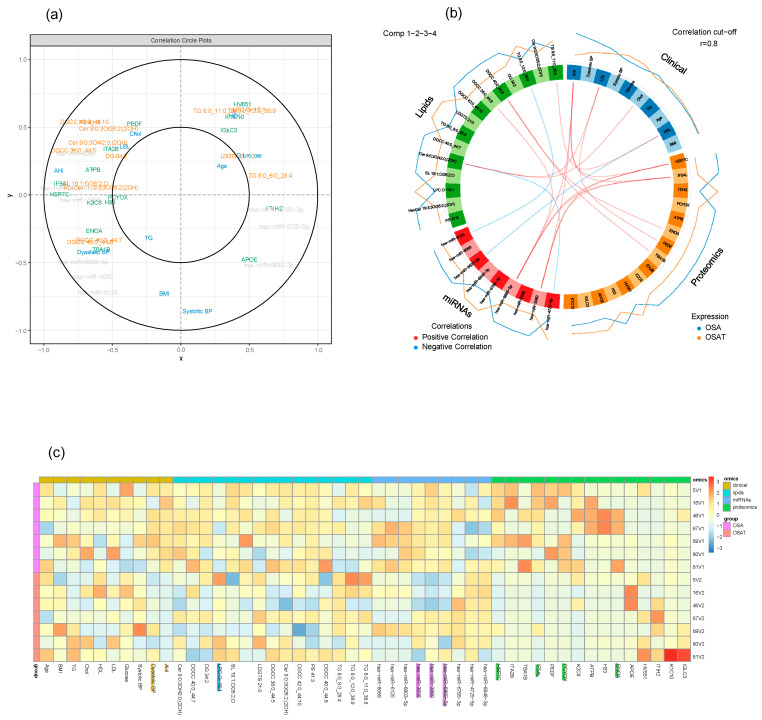
Graphical representation of a multi-block analysis performed on OSA and OSAT. (**a**) Variable plot highlighting the contributions for OSA clinical data, exosome cargo including lipids, proteins, and miRNAs. Markers in the outer circle are more significant and contribute more to separating the two conditions. Those in the inner circle are less significant than ones in outer circle. (**b**) Circos plot depicting the strongest correlation biomarkers in the multi-omic biomarker panel in OSA and OSAT. Circos plot of clinical, lipids, proteins, and miRNAs showing Spearman’s correlation analysis (*p* < 0.05) between OSA and OSAT multi-omics, classified based on the superclass. (**c**) Heatmap clustering of the variables (clinical data, lipids, proteins, and miRNAs) to represent the muti-omic profiles for the 7 samples. Red indicates a positive correlation, and blue indicates a negative correlation. The variables were selected by applying DIABLO to cellular frequency, gene, and metabolite module datasets depicted using a Circos plot. The variables indicated in the ideogram are connected with either red or blue to other variables if the correlation is either positive or negative. Only correlation above a certain threshold is depicted (r = 0.8). The lines around the ideogram are drawn by connecting the average expression value of a given variable for a certain phenotypic group.

Using multi-block analysis, we identified variables from each block that are involved in discrimination according to the OSA treatment. The similarities between OSA and OSAT subjects were assessed by several graphical block outputs with 10 clinical features (age, BMI, Chol, TG, HDL, LDL, AHI, glucose, and systolic and diastolic blood pressure), 72 lipid features, 16 proteomic features, and 13 miRNA features based on volcano plots (Figure S9). Among the 12 subjects of OSA and OSAT, 7 subjects had complete comprehensive exosome cargo information including lipids, proteins, and miRNAs (Figure S9). The interpretation of multi-block data requires several graphical outputs. Some of them are presented in Figure S9 for the sparse version. The sample plots for each dataset including clinical data, lipids, proteins, and miRNAs are shown in Figure 7a. Using the results of the sparse version of the multi-block analysis, we identify variables from each block that are mainly involved in discrimination according to the OSA condition. For instance, variables located within the outer circle are most important and contribute more in terms of differentiating between all omics datasets for the samples of OSA and OSAT (Figure 7a). The correlation circle plot shows the contribution of each variable to each component (x and y). The variables that are closer to the outer circle are more important than those that fall in the inner circle. For example, in Figure 7a, clinical variable AHI has a negative contribution on component x, and Systolic BP clinical variable has negative contribution on component y. The clinical variable AHI, miRNA variables hsa-miR-6869-5p and hsa-miR-4725-5p, and lipid variables HSP7C and IF5AL are very important and contribute highly towards discriminating the OSA and OSAT samples on component X. The clinical variable Systolic BP contributes highly towards discriminating the OSA and OSAT samples on component y. All the variables located between the two circles contribute towards discriminating the samples to some lesser or greater degree. The individual variables for each omics dataset can also be seen in Figure S9, and the loading weights can be visualized in the contribution plot (Figure S10), where most of these differentiating features can be seen at the bottom of the plot. In an integrative study, all blocks acquired for each sample can be analyzed together through a multi-block analysis, and a Circos plot can be generated to help interpret the results of this multi-block approach (Figure 7b). The Circos plot represents the correlations greater than 0.8 between lipid, clinical, proteomic and miRNA variables. The internal connecting lines show the positive (red) and negative (blue) correlations. The outer lines show the expression levels of each variable in each sample group (OSA and OSAT). The variables are sorted first according to their block and then depending on their importance in discrimination. The relationships are positive and concern a few variables from each block. The selection of variables is valuable information for the biologist in that it allows to focus on this smaller selection for validation and draw biological conclusions from it. Relevance networks can also be viewed as an initial step in modelling since they mimic biological networks and provide clues to address inference network issues through further dedicated experiments. Another way to display the results is presented in Figure 7c. The heatmap shows the multi-omic molecular signature expression for each sample. It represents samples in rows (indicated by their group on the left-hand side of the plot) and variables in columns (indicated by their data type at the top of the plot). The variables have strong contributions are highlighted at the bottom, along with their labels. Clinical variables are highlighted in yellow, lipid variables in blue, miRNA in purple, and proteomics in green. The heatmap highlights the profiles of selected variables for each of the OSA and OSAT samples and omics data types, with both positive and negative relationships plotted in Figure S11.

## 3. Discussion

In this study, we conducted a multi-omic exploration of circulating exosomal cargo by integrating lipidomics, proteomics, and small RNAs (miRNAs) from exosomes derived from OSA patients at the time of their diagnosis and then following long-term adherent CPAP treatment. Our aims were twofold: (a) to study the effects of exosomes on endothelial barrier integrity, wound healing, and tube formation, and (b) to determine differentially expressed exosome cargos, including lipids, proteins, and miRNAs. These two parallel aims enabled the creation of a network-based model for a better understanding of the biochemical alterations caused by OSA and how they connect with each other from a systems biology perspective. Multi-omic analyses further enabled the identification of substantial interactions between specific phenotypic clinical characteristics and the three omic-based explorative approaches implemented herein. For example, we found that the cholesterol metabolism (has04979) pathway is associated with lipid classes in OSA patients. Furthermore, our data suggest that a multi-omic integrative approach might be useful in understanding how exosomes function, their origin, and their potential clinical relevance, all of which merit future exploration in the context of relevant phenotypic variance. Thus, exosome profiling may reveal pathological events occurring at the cellular and systemic levels in OSA patients, pinpointing deregulated molecular pathways and possibly therapeutic targets. In fact, the current unavailability of disease-modifying therapies for OSA is a constant reminder of the need to better understand its molecular mechanisms. Developing more effective/etiology-driven treatments may be possible by understanding the molecular changes in OSA. Comprehensive analysis of multi-omic data should provide useful insights for discovery of new biomarkers and identification of therapeutic targets.

OSA is a highly prevalent disease that imposes a myriad of adverse consequences, among which cardiovascular morbidity is particularly prominent [45]. Chronic OSA is becoming increasingly prevalent due to factors such as its potential links to metabolic syndrome or an increased awareness of OSA patients, resulting in more diagnoses [46,47]. More than 900 million adults have been affected by OSA globally, with about two-fifths in the moderate to severe category [1]. In general, OSA prevalence ranges from 9% to 38%, with males (13% to 33%) more likely to be affected than females (6% to 19%) [46]. OSA can adversely affect the hypoxia-reoxygenation system and sleep cycle, increasing inflammation, oxidative stress, endothelial dysfunction, and sympathetic activity. As a result, adverse cardiovascular events are more likely to occur [48]. Studies have shown that OSA can result in systemic and local inflammation, and this inflammation can trigger the impairment of vascular endothelial cells and further modify the structure and function of vessels, leading to endothelial dysfunction [49]. Endothelial dysfunction is a key factor in the development of various end-organ morbidities, such as CVD and metabolic dysfunction [49,50]. Poor adherence to CPAP treatment by adults with OSA is a common issue [51], and a recent study suggested that CPAP treatment does not substantially improve metabolic derangements in an unselected OSA population, but the effect may be higher in specific subgroups of OSA patients [52]. We present for the first time a comprehensive study that incorporates clinical phenotype, bioinformatics, multidimensional network analysis, and state-of-the-art systems biology in an effort to integrate the circulating exosome cargo in OSA patients before and after treatment into a cogent and evidence-based guide of potential pathophysiological mechanisms driving this chronic condition. This work provides an important new basis for hypothesis generation and mechanistic insights into OSA pathobiology and allows for the identification of novel diagnostic and pharmacological targets for early diagnosis and treatment of OSA. Several studies have suggested that OSA-induced repeated hypoxia may play a role in the pathophysiology of cardiovascular diseases by inducing inflammatory responses through increasing cytokine and adhesion molecule levels [53,54]. Together, our multi-omic assessment of OSA-derived exosomes provides several new insights. For example, alterations of exosome lipidomic, proteomic, and miRNA profiles in OSA are associated with a combination of temporal changes that may not only enable specific disease phenotype detection but may also drive recognition of mechanisms leading to specific end-organ morbidity.

Exosomes are abundantly found in all bodily fluids, and their cargo is continuously influenced by ongoing physiological and pathological events, making them outstanding diagnostic markers. In addition, exosome cargo can provide unique information regarding mechanisms underlying the disease, as well as enabling prediction of clinical outcomes in a large number of disorders [55,56,57]. Most studies to date have focused on characterizing plasma exosomes with the aim of exploring their role in various pathogenic processes at the transcriptomic, proteomic, metabolomic, lipidomic, and genomic levels. However, integrative multi-omic studies of exosomes have been relatively scarce, and yet they have afforded uniquely valuable insights into the mechanisms underlying disease and into some of the biological roles played by different kinds of exosome cargo in the context of disease diagnosis and prognosis. Using the latter multi-omic approach, the current study detected potential alterations in several biological systems (e.g., hemostasis, immune system, metabolism, etc.) that provided confirmatory network cross-correlations across the various Omics utilized herein. We surmise that exosomes provide an additional level of biological complexity, as they play a key role in cellular communication and mediate specific signals to cells and tissues, thereby reflecting not only specific processes within affected cells, but further conveying unique cellular responses to both neighboring and distant cellular systems. We therefore postulated that analysis of plasma exosomes by multi-omic approaches may provide unparalleled insights into the involvement of concurrent pathogenetic mechanisms that, by virtue of their inter-individual differences, may facilitate a more personalized and precise approach to each patient. Thus, even if the functional properties of exosomes from each OSA patient led to dysfunction of naïve endothelial cells, the conglomerate of exosome elements potentially contributing to such common functional-deficit end results will differ and facilitate improved understanding of the specific contributors to morbidity (e.g., endothelial dysfunction) in each patient.

### 3.1. Functional Effects of Circulating Exosomes on Naïve Endothelial Cells

Vascular endothelial dysfunction, characterized by imbalanced vasoconstrictive and vasodilatory molecules, is the earliest sign of vessel lesions preceding clinically obvious cardiovascular complications in OSA [18,58,59]. Circulating exosomes are constantly in contact with endothelial cells, and they regulate endothelial cell proliferation, apoptosis, and migration, thus regulating vascular function [60]. Exosomes are produced by all cells, and endothelium is a rich source of exosomes that have access to the main circulation, thereby potentially impacting local and distant tissue function [61]. We showed that plasma-derived exosomes from OSA patients impair endothelial adhesiveness and permeability [24,62,63], which may directly or indirectly trigger or exacerbate cellular endothelial injury, possibly via oxidative stress-related pathways. Furthermore, we previously showed that circulating exosomes contribute to the senescence of endothelium in OSA, and are amenable to improvements, at least in part, after treatment of OSA with adherent CPAP [39]. In obese children or in children suffering from OSA who manifest evidence of endothelial dysfunction, plasma exosomes induce marked in vitro and in vivo functional and structural alterations in naïve endothelium that are mediated by selective components of the exosomal miRNA cargo [63]. Of note, such effects are not present when similar experiments are conducted among children with obesity or OSA but without evidence of endothelial dysfunction. Plasma-derived exosomes in otherwise healthy subjects exposed to 4 days of intermittent hypoxia mimicking OSA are constitutively altered in their miRNA cargo and exhibit the ability to induce endothelial dysfunction in vitro [64]. We further demonstrated that such properties are reversed upon normoxic recovery [24]. In patients suffering from the obstructive hypoventilation syndrome (OHS), the most severe form of sleep-disordered-breathing, we found that circulating exosomes contributed to the induction and propagation of OSA/OHS-related endothelial dysfunction (i.e., increased permeability and disruption of tight junctions along with increased adhesion molecule expression and reduced endothelial nitric oxide synthase expression) and promoted increased monocyte adherence [65]. Our current findings expand on these previous studies and illustrate an expanded repertoire of adverse functional consequences imposed on naïve endothelial cells when subjected to exosomes from OSA patients.

### 3.2. Multi-Omic Analysis

#### 3.2.1. Lipid Cargo of Exosomes

Lipids represent one of the most important components of exosomes, with important structural and regulatory functions during exosome biogenesis, release, targeting, and cellular uptake [66]. For example, lipids are essential elements that have been found in all cell types and are abundantly distributed in exosomes. The lipid profiles of exosomes have been reported for several cell lines and biological fluids, including urine, plasma, and serum [67]. The lipidomic characteristics of OSA patients have been previously reported, and widespread alterations across the spectrum of lipid classes were detected in patients with OSA when compared with controls [68,69], with some of these findings potentially pointing to identification of the biochemical mechanisms affecting lipid metabolism in OSA, or to the discovery of novel biomarkers, and further to the evaluation of treatment efficacy [70]. In this study, we found that exosomes derived from OSA subjects are enriched in multiple lipid classes, including glycerolipids (51.4% ± 1.15), phospholipids (38.41% ± 1.74), saccharolipids (6.51% ± 0.92), and sphingolipids (3.68% ± 0.14). We identified several lipid classes in exosomes which may play regulatory functions. For example, ceramide is a sphingolipid which is one of the most important lipids in exosome biogenesis because of its apparent capacity to trigger ESCRT-independent processes and induce spontaneous membrane invagination [71]. Ceramide is synthesized from SM after the removal of a phosphocholine moiety by sphingomyelinases, and the spontaneous budding of ceramide-containing membranes is attributed to its cone-shaped structure, which facilitates the negative curvature of the membrane [72]. In addition, phospholipids that appear to play important regulatory functions during exosome biogenesis include phosphatidylinositol 3-phosphate and phosphatidylinositol 3,5-biphosphate, which seem to regulate the exosome formation, release, and cargo sorting [66].

To assess the potential of exosomal lipid signatures in OSA, we performed ROC analysis of OSAT vs. OSA for multiple lipid classes. Our data suggests multiple lipid profiles for each of the sub-classes identified under the AUC. As an illustrative example, the highest AUCs for glycerolipids were TG 8:0_9:0_38:4 and TG 54:4|TG 18:1_18:1_18:2, with additional biomarkers emerging for phospholipids, saccharolipids, and sphingolipids. We suggest these specific lipid subsets may serve to identify a circulating plasma lipidomic signature that may be further refined to demarcate between relevant OSA clinical phenotypes. Since the extant research focused on exosomal lipids in the context of vascular dysfunction is limited, further extrapolation of our findings to parallel studies is precluded. However, serum lipidomic profiling revealed dysfunction of phospholipid metabolism in subclinical coronary artery disease [73]. There was also a trend of higher levels of LPC(18:0) and LPC(22:6) and lower levels of LPC(16:0), LPC(16:1), LPC(18:1), LPC(18:2), LPC(20:3), and LPC(20:4) in severe coronary calcification. These results were supported by similar findings that showed lower levels of LPC (16:0), LPC(18:2), and LPC(20:4) associated with CVD [74,75]. Thus, changes in lipid composition apparently increase the exosomes’ ability to fuse with neighboring cells [76]. Many proteins and pathways have been linked to exosome export, including the ceramide pathway. For example, ceramide has been shown to facilitate the formation of endosomal vesicles, and the export of specific miRNAs to exosomes [72,77]. Understanding lipid heterogeneity within exosomes could prove critical toward understanding internalization mechanisms and signaling events which may not be captured by more commonly used proteomic and nucleic acid characterization techniques [78,79]. Indeed, the main role of lipid components in exosomes is to regulate exosomal sorting of miRNAs and proteins [80]. Lipid dysmetabolism in OSA reflects alterations in phospholipid biosynthesis, steroidogenesis, and fatty acids. This may influence cell membrane formation, augmenting lipid uptake, atherogenesis and inflammation [81]. The use of metabolomic and lipidomic strategies for selecting potential biomarkers for OSA was explored [68,82].

#### 3.2.2. Exosome Proteins

We performed an extensive investigation of the exosome protein cargo via quantitative proteomic approaches in patients with OSA before and following adherent long-term therapy. Previous studies have used proteomics of different exosomes subtypes to begin to evaluate the specificity of protein markers classically used to define exosomes and other exosome subtypes [83,84], since plasma- and serum-derived exosomes are considered liquid biopsies for disease-associated changes because exosomes are shed into the blood from the tissue of origin. OSA and OSAT have different abundance levels for a selected number of proteins. These altered proteins were distinctive between OSA and OSAT based on hierarchical clustering, PCA, and Pearson Correlation Analysis (PCA). To further delineate the functional relevance of the altered proteins, we conducted GO and pathway analysis (KEGG and Reactome) and summarized four sub-categories of GO BP terms and KEGG/Reactome pathways based on the enrichment results. We analyzed these proteins using GO enrichment analysis, and we selected the top 10 genes for biological process (PB), cellular component (CC), and functional process. Many of the enriched biological processes found for OSA exosome proteins are involved with biological processes, immune response, and cellular processes. Some of the proteins that were identified have been implicated in well-established morbidities associated with OSA, such as coronary artery disease (PLAT, APOB, APOA1, APOE, PCSK9, LPA), lipid metabolism disorder (APOC3, APOB, APOA1, APOE, LCAT, PCSK9, LDLR), vascular disease (PLAT, SERPINE1, APOB, APOA1, APOE, PCSK9, LPA), atherosclerosis (APOB, APOA1, APOE, LPA), and neurodegenerative disease (APOE, SORL1, APP, BACE1, MAPT, TREM2, VLDLR). In addition, we evaluated the diagnostic probability of OSA and the ROC for each of the 17 differentially expressed proteins ranging from 0.6 to 0.9. Of these 17 proteins, we highlighted only the highest proteins with AUC values. For example, HSP7C was the highest, followed by ATPB and IF5ALS, based on 80% specificity and sensitivity values. Thus, proteomic analyses have provided information regarding cholesterol transfer activity, enzyme regulator activity, and phospholipid binding that are relevant to the pathophysiology of deleterious effects induced by the presence of OSA. Proteomic analyses of circulating exosomes derived from OSA represent a promising approach to the elucidation of cell–cell communication and the discovery of putative biomarker candidates for OSA diagnosis and treatment. A proteomic approach has been performed to detect protein profiles of serum extracellular microvescicle proteins in an intermittent hypoxia (IH) rodent model [85]. Furthermore, proteomic analysis using high-resolution and high-throughput mass spectrometry has been reported with OSA [86,87,88].

#### 3.2.3. Exosome miRNAs

MiRNAs play a key role in exosomes due to their ability to protect against degradation and increase stability. Since one miRNA can target and regulate hundreds of genes (mRNAs), miRNAs have also gained considerable attention as biomarkers or putative indicators of mechanistic pathways in disease states. Furthermore, differential plasma miRNA profiles have been described for many diseases, including fatty liver [89] and atherosclerosis [90,91]. Dysregulation or altered miRNA expression/function has been implicated in diabetes [92] and cardiovascular disease [93,94]. Furthermore, the role of miRNAs in cholesterol homeostasis and lipid metabolism has been the focus of multiple studies [95,96]. We have previously identified a cluster of exosomal miRNAs diverging in normal dipping blood pressure (NDBP) and reverse dipping blood pressure (RDBP) in the context of OSA [62], i.e., underlying different clinical phenotypes despite similar OSA severity based on polysomnographic criteria. Of note, 10 of the miRNAs in that study were the same as in the current study, namely hsa-miR-6089, hsa-miR-933, hsa-miR-4725-5p, hsa-miR-33b-3p, hsa-miR-6508-5p, hsa-miR-1238-3p, hsa-miR-1228-3p, hsa-miR-6797-3p, hsa-miR-6069, and hsa-miR-4665-3p. Such striking similarities further buttress the congruence of the pathophysiological pathways likely involved in downstream effects at the organ and cellular level. Indeed, miRNAs, messenger RNAs, and proteins contained in exosomes can influence the development of atherosclerosis or angiogenesis in the context of peripartum cardiomyopathy [97]. Furthermore, several miRNAs have been implicated in OSA pathophysiology, including miR-664a-3p, miR-92a, and miR-1254 [98,99,100]. In addition, miR-210 concentration was higher in patients with OSA than in matched control subjects, and the AHI of OSA subjects was positively correlated with miR-210 concentration among individuals with OSA, thus suggesting a critical role of miR-210 in OSA pathophysiology [101]. OSA was also associated with dysregulation of several novel non-coding RNAs, including lncRNA MRPL20-AS1, miRNA-1254, and miR-320e [102,103].

### 3.3. Data Integration

In the various paragraphs above, we have discussed each of the omics findings separately. However, the unique value of multi-omics resides in the ability to effectively generate biologically relevant data integration approaches that can identify novel biomarkers and gain profound insights into biological mechanisms from different experimental sources [104]. Exosome cargos are primarily composed of proteins, lipids, and miRNAs and play multiple simultaneous roles throughout the human body [105]. Exosomal cargos that jointly explore miRNAs, proteins, and lipids have been reported as promising biomarkers in pancreatic cancer [106], prostate cancer [107], and stroke [108]. Here, we have identified a panel of constitutive elements within exosome cargos and corresponding downstream molecular pathways that exhibit strong associations with OSA clinical outcomes. To achieve such goals, we used correlation network analysis to identify and visualize relationships between key features from omics datasets. Thus, this study aims to introduce exosomes in a multi-omics context and to provide a perspective on their potential landscape applicability to a disease such as OSA. Similar approaches have recently been applied to Alzheimer’s disease, whereby investigators detected unique biomarkers by integrated analysis of 1000 proteins, 594 lipids, and 105 miRNAs derived from microglia [109]. Multidimensional cargo carried by exosomes in circulating blood may therefore reflect pathophysiological processes occurring within their source cells and tissues. The clinical variable AHI, miRNA variables hsa-miR-6869-5p and hsa-miR-4725-5p, and lipid variables HSP7C and IF5AL are very important and contribute highly towards discriminating the OSA and OSAT samples on component X, and the clinical variable Systolic BP contributes highly towards discriminating the OSA and OSAT samples on component y (Figure 7a). There are several advantages to studying exosome cargo, and in particular miRNA, in circulating exosomes rather than total miRNAs in plasma: (1) Exosomes provide relevant information on patient status and may offer prognostic information on a broad range of diseases [110]. (2) Through the use of circulating exosomes, real-time data can be gathered from the different cells involved in the pathological process (e.g., injured cells, immune cells, and metastatic cells) [111]. (3) miRNAs are more stable in exosomes, and their accessibility through biological fluids makes them an attractive alternative as a minimally invasive diagnostic test, a liquid biopsy [112]. (4) Exosomes transfer miRNAs from donor to recipient cells, regulating gene expression locally and distantly during both physiological and pathological processes. Due to this transfer capability, exosome-associated miRNAs can serve as diagnostic and prognostic biomarkers and therapeutic agents [28,30,113].

Several limitations in this study merit mention. Among them is the fact that only male participants were included, and the sample size was small. The age range of the patients was wide and therefore would not allow for differentiating between younger and aging patients despite the differences in the risk of complications of the disease related to age and sex differences [114]. Similarly, generalizability to other ethnic or racial groups or to anthropometrically defined patient subsets would also be desirable. However, a major strength of the study is that we used the same subjects before and after adherent CPAP treatment for 1 year. Of note, the specific role played by each of the differentially expressed elements in the context of the various omics implemented herein was not sought and must await future focused studies. Our findings highlight the importance of exosomes in mediating OSA disease and provide insights into the molecular pathophysiology of OSA while suggesting avenues for the prevention of health-to-disease transitions. The exosomes derived from OSA disrupt the endothelial barrier integrity and angiogenesis, as opposed to OSAT, in vitro. Exosome cargos including specific proteins, miRNAs, and lipids are linked to the pathophysiological functions of exosomes, may lead to the discovery of biomarkers, and may also facilitate the unraveling of molecular mechanisms underlying cargo-sorting and biogenesis of exosomes in a disease such as OSA. We provided a bio-signature feature list containing lipids, proteins, and miRNAs, and clinical data which discriminate between OSA and OSAT subjects. These multi-omic characteristics in the OSA group are strongly associated with the known evidence on the pathogenesis of the disease. Developing an integrated molecular classification should improve diagnostic classification, risk stratification and assignment of molecular, disease-specific therapies to improve the care of patients with OSA from a personalized medicine perspective.

## 4. Materials and Methods

### 4.1. Subject Characteristics

Human studies were conducted at the Sleep Clinic of the Hospital Universitario Miguel Servet, a large teaching hospital in Zaragoza, Spain, as part of the EPIOSA study (NCT02131610) as previously described [115]. This prospective study included 18–60 year-old patients with polysomnographically-diagnosed OSA who went on to complete CPAP titration and subsequent treatment following a validated protocol [115]. The initial 12 subjects with OSA at baseline (OSA) and after 12 months of adherent CPAP treatment (6.16 ± 0.88 h/night throughout) (OSAT) were selected into the present study. Data from all sleep studies were scored using American Academy of Sleep Medicine guidelines [116] by trained personnel that were blinded to the aims or nature of the study. At baseline and at every follow-up visit, smoking status was evaluated with co-oximetry and questionnaire. Blood samples were drawn at baseline upon diagnosis of OSA and from the same subjects 12 months later following adherent CPAP treatment (OSAT) using a 21-G butterfly needle into ethylenediaminetetraacetic acid (EDTA) (PreAnalytix, GmbH, Switzerland). Biochemical tests were measured using serum glucose; triglycerides, total cholesterol, and high-density lipoprotein cholesterol were measured by spectrophotometry (Chemical Analyzer ILAB 650, Instrumentation Laboratory). Plasma was separated by centrifugation and stored at −80 °C.

### 4.2. Exosome Isolation and Characterization

Plasma exosomes were isolated and characterized as previously described [7,24,63]. The isolated exosomes were subsequently quantified and characterized following MISEV2018 guidelines [117]. Transmission electron microscopy (TEM) was used to determine exosome size as previously described [39,62,63]. Exosome quantifications were determined using NanoSight, NS300, (Malvern Panalytical, Malvern, UK) equipped with a high sensitivity sCMOS camera, 531 nm laser, and automatic syringe pump [26]. Exosome aliquots were fixed in 2% paraformaldehyde; 5 μL of exosome suspension was then applied to each formvar/carbon-coated 200 mesh nickel grid and allowed to adsorb for 2 min. Grids were incubated with 30 μL drops of 2% uranyl acetate and examined by electron microscopy [64]. The samples were washed with distilled water seven times (2 min each), and then they were viewed under a FEI Tecnai F30 Twin (Atlanta, GA, USA) transmission EM to measure the size of the isolated EVs [64].

### 4.3. Exosome Markers Using Flow Cytometry

To analyze for selective sub-populations of exosome surface markers, exosomes were incubated with Exo-Flow™ kits (System Biosciences, Mountain View, CA, USA) and then subjected to FACS analysis (FACSCalibur, BD Biosciences, San Jose, CA, USA) as previously described [64]. Exosomes were incubated with commercially available magnetic beads of 9.1 nm diameter that incorporated different exosome markers, including tetraspanins (#EXOFLOW150A-1, CD63, and CD81). In the FACS, 25,000 events were acquired and then analyzed using FlowJo Software 2.9.0 (Tree Star, Inc., Ashland, OR, USA). Two negative controls were also carried out, with negative #1 (all reagents without antibodies and no EVs) and negative #2 (all the reagents and beads but without EVs). The average of MFI for negative #1 was used to normalize the samples with and without exosomes.

### 4.4. Human Endothelial Cells and Exosome Uptake

Human microvascular endothelial cells, dermal (HMVEC-d), were purchased from Lonza (catalog # CC-2543; Lonza, Alpharetta, GA, USA) [26,39]. Cells were grown in endothelial growth medium (EGM-2-MV; Alpharetta, GA, USA) supplemented with 5% fetal bovine serum, FBS, (Life Technologies, Grand Island, NY, USA), and further incubated at 37 °C in a cell culture incubator. The cells were trypsinized and centrifuged at 250× *g* for 5 min, diluted, and re-plated at appropriate densities. All cells were used before passage 4. HMVEC-d-confluent cell monolayers were grown on 12 cover slips for 24 h in EGM-2-MV medium containing 5% fetal bovine serum (FBS), after which cells were washed with medium containing depleted FBS (System Biosciences, Mountain View, CA) Labeled exosomes were added to cover slips, and cells were fixed with 4% (*w*/*v*) para-formaldehyde in 1X PBS for 15 min at room temperature, then washed again with PBS. The cell membranes were permeabilized by incubation with 0.25% (*v*/*v*) Triton-X-100 in PBS for 10 min.

Exosomes were labeled with a lipophilic fluorescent dye, PKH67 (Sigma, #PKH67-GL-1KT, St. Louis, MO, USA) and unbound dye was removed using Vivaspin 20, 3 kDa MWCO centrifugal filters (Sigma, #Z629456), according to the manufacturer’s protocol. The pellets were suspended in 1× PBS buffer and filtered, and the labeled EVs were placed on confluent coverslips of HMVEC-d (Sigma-Aldrich, Millipore, CA, USA) (30 µg/mL) for 24 h in a cell culture incubator at 37 °C. PKH67 colors were monitored for delivery into target cells using a Leica SP5 Tandem Scanner Spectral 2-photon confocal microscope (Leica Microsystems, Buffalo Grove, IL, USA) with a 63× oil-immersion lens. As negative control, PKH67 were prepared and added to each cell with all reagents, but no exosomes, to monitor unincorporated dyes. Cell nuclei were visualized by staining with DAPI at a concentration of 1 μg/mL in PBS (Life Technologies, Carlsbad, CA, USA) at room temperature for 5 min [118,119,120]. Images were captured with a Leica SP5 Tandem Scanner Spectral 2-photon confocal microscope (Leica Microsystems, Inc., Buffalo Grove, IL, USA) with a 63× oil-immersion lens.

### 4.5. Endothelial Cell Barrier Integrity

Real-time change in trans-endothelial monolayer electrical resistance was measured using an ECIS system. The ECIS assays were conducted using 8-well ECIS arrays (PC; 8W10E) via the ECIS-Z station. The arrays (8W10E) were treated with 10 mM l-cysteine (Sigma-Aldrich, Millipore, St. Louis, MO, USA) followed by coating with Collagen Type II (Sigma-Aldrich, Millipore, St. Louis, MO, USA) as previously described [62]. A total of 50,000 cells were plated and grown to confluence into ECIS arrays as a single confluent monolayer. ECIS assessments were performed using multiple frequency/time (MFT) options to record continuous impedance changes over a broad spectrum of frequencies. When impedance signals stabilized and therefore indicated that a confluent monolayer and a functional barrier had formed, EVs were added in duplicated wells and placed into the ECIS instrument for continuous monitoring for up to 24 h. As cultured cells adhered and spread on the electrode surface, the impedance changed, and such changes over time served as a measure of the disruption of the endothelial cellular junction. Control reference values were established by using culture medium (500 μL/well) alone, and then compared with the values recorded when electrodes were covered with a monolayer of cells in 500 μL medium.

### 4.6. Wound-Healing Assay

ECIS was used to monitor the recovery of HMVEC-d cells in a real-time fashion after wound healing [121,122]. HMVEC-d were seeded on 8W10E gold electrode arrays, and cells were wounded by applying a burst of high-intensity electrical current while they were maintained in a humidified 5% CO_2_ incubator at 37 °C. The arrays (8W10E) were treated with l-cysteine and with Collagen Type II, as described above. ECIS was performed while recording the multiple frequency/time (MFT) option to evaluate impedance changes over a broad spectrum of frequencies. Before starting ECIS measurements, 300 µL medium containing 5% depleted bovine albumin serum (BAS) was placed in each well and allowed to stabilize for 30 min, after which 200 µL of cell suspension (5 × 10^5^ cells per mL) was added to each well. After cell inoculation, the wells were incubated for 24 h, and once they reached confluence, wounding was carried out with the integrated electrical field module (3500 µA, 20 s at 48 kHz) for a total time of 5 min. Exosomes (30 μg/mL) were applied to the designated wells. The healing process was monitored continuously as cells migrated and proliferated onto the electrode. Control reference values were established by using culture medium (500 μL/well) alone, and then compared with the values recorded when electrodes were covered with a monolayer of cells in 500 μL medium. Data were acquired with a frequency of 4000 Hz. Resistance values were collected and normalized to each well’s value at *t* = 0. The healing process was monitored continuously as cells migrated and proliferated onto the electrode.

### 4.7. Angiogenesis Tube Formation Assay

The Angiogenesis Tube Formation Assay was conducted according to the manufacturer’s instructions (# 3470-096-K, Trevigen, Gaithersburg, MD, USA). Briefly, 50 μL Basement Membrane Extract (BME) (Cat#: 3433-005-01) were prepared on ice and further were incubated at 37 °C for 1 h. HMVEC-d (10 × 10^3^) cells were seeded and, immediately following seeding, exosomes from OSA or OSAT (30 μg/mL) were added, supplemented with endothelial cell growth medium-2 (EGM-2, Lonza) in depleted FBS, as previously described [26]. Microscopic images were captured using a Nikon Eclipse Ti microscope equipped with a 10× phase-contrast objective (Nikon Instruments, Melville, NY, USA). Tube formation was monitored and imaged with a Nikon Ellipse T*i* microscope (Nikon Inc., Melville, NY, USA). The relative vessel area, tube length, relative average vessel length, and relative total number of junctions were counted by the Angiogenesis Analyzer plugin [123] of ImageJ (version 1.53, Bethesda, MD, USA). The mean of the total tube length per total area imaged (μm tube/mm^2^) was calculated for each well. The experimenter was blinded to the experimental groups throughout the period of analysis. The tubular structures were observed and monitored automatically every 30 min for 48 h using a real time cell recorder microscope for cells not treated with exosomes, cells treated with OSA exosomes, and cells treated with OSAT exosomes.

### 4.8. Exosome Lipidomics

Lipids were isolated from 50 µg of exosomes from each OSA and OSAT sample, as previously reported [124]. Briefly, 185 µL of chloroform (CHCl3) containing docosanol (10.0 μg/mL) and 220 µL of methanol were added to 50 µL of the sample, vortexed for 1 min, and placed on an orbital shaker for 10 min. Then, 185 µL of CHCl3 with docosanol and 200 µL of water were added to the mixture, vortexed for 1 min, and centrifuged at 3000× *g* for 15 min to separate into two layers. The lower CHCl3 layer was dried under nitrogen gas. The dried lipids were re-suspended in 100 µL of CHCl3 and methanol (1:1, *v*/*v*) and analyzed on a liquid chromatography–mass spectrometry (LC-MS, Bruker maXis impact quadrupole-time-of-flight mass spectrometer coupled to a Waters ACQUITY UPLC system). Separation of lipids was achieved on a Waters C18 column (2.1 × 150 mm, BEH C18 column with 1.7-um particles) using a linear gradient and mobile phase A (water containing 0.1% formic acid and 2 mM ammonium format) and B (Methanol containing 0.1% formic acid and 2 mM ammonium formate). The gradient condition for B increased from 5% to 70% over 5 min, then to 95% over 3 min, held at 95% for 3 min, then returned to 5% for equilibrium. The flow rate was 0.56 mL/min, with a column temperature of 60 °C. Full scan mass spectral data were collected from *m*/*z* 100 and 1500, and MS/MS spectral data were acquired using auto MS/MS. Mass spectra were auto-calibrated using sodium format after data acquisition. The data were processed with Bruker Metaboscape software 4.0 to extract mass features and lipids identified by matching their MS/MS spectra against in silico MS/MS spectra from LipidBlast [125]. All the data were normalized to sum, log-transformed, and Pareto-scaled. All reported lipid identifications follow the nomenclature of LIPID MAPS Lipid Classification System

### 4.9. Exosome Proteomics

Proteins were extracted from 12 OSA and OSAT exosome samples using a RIPA lysis buffer with protease inhibitor cocktail (Sigma-Aldrich), and total protein content was determined using a Pierce BCA protein assay kit (Thermofisher Scientific, Berkeley, MO, USA) according to the manufacturer’s instructions. The protein concentration from OSA and OSAT were determined using a QuickStart Bradford assay (BioRad, Hercules, CA, USA). Briefly, 75 µg of exosome proteins from each sample were added to cold acetone and kept overnight at −20 °C. Protein-exosome acetone pellets were washed with 80% acetone in water and re-suspended in 25 µL urea buffers (6 M urea, 2 M thiourea, 100 mM ammonium bicarbonate, pH 8.0). Proteins were digested for 4 h with 0.75 µg LysC. The samples were diluted 10-fold for overnight 0.75 µg trypsin digestion. Peptides were purified using Pierce 100 µL C18 tips and lyophilized. Lyophilized peptides were re-suspended in 25 µL of solvent (5% acetonitrile, 0.1% formic acid) to approximately 3 µg/µL and stored in an autosampler at 7 °C. Peptides were analyzed as follows: 3 µL were injected onto a C8 trap column (Thermo Fisher Scientific, Berkeley, MO, USA), µ-precolumn—300 µm i.d. × 5 mm, C8 Pepmap 100, 5 µm, 100 Å) and separated on a 20 cm long × 75 µm inner diameter pulled-needle analytical column packed with Waters BEH-C18, 1.7 µm reversed phase resin. Peptides were separated and eluted from the analytical column with a gradient of acetonitrile at 300 nL/min. The Bruker nanoElute system was attached to a Bruker timsTOF-PRO mass spectrometer via a Bruker C2aptiveSpray source. Initial gradient conditions were 2%B (A: 0.1% formic acid in water, B: 99.9% acetonitrile, 0.1% formic acid), followed by a 20-min ramp to 17%B. PASEF 70 min LCMS data were acquired on a Bruker timsTOF, and data searched against Uniprot-Human using PEAKS. A pair-wise comparison was done using PEAKSQ. This quantitation is based on precursor (peptide) intensity and is corrected for mass and retention time matching. Protein–protein interaction networks were performed using String software (Version 12.0) [43].

### 4.10. Exosome miRNAs

Total RNAs, including miRNAs, were isolated from exosomes derived from 12 OSA plasma samples and corresponding OSAT, using miRNeasy Serum/Plasma Mini Kit columns following the manufacturer’s instructions (Qiagen, Valencia, CA, USA), as previously described [63]. Total RNAs were quantified on a Nanodrop 2000 (Ambion, Austin, TX, USA), and RNA quality and integrity were determined using the Eukaryote Total RNA Nano 6000 LabChip assay (Agilent Technologies, Santa Clara, CA, USA) on the Agilent 2100 Bioanalyzer. The quality of miRNAs was determined using an Agilent Small RNA Kit [63]. The miRNA expression analyses were performed using human miRNA microarray for one-color technique (Agilent Technologies, Santa Clara, CA, USA) consisting of 60-mer DNA probes synthesized in situ that represent 2529 human mature miRNAs derived from miRbase version 21:0, and 39 viral miRNAs.

Total RNA (100 ηg) was labeled and hybridized on a microarray (miRNA complete labeling and hybridization kit) and afterwards scanned using DNA Microarray Scanner (Agilent Technologies, Santa Clara, CA, USA) [63]. Total RNA, including enriched miRNA, was dephosphorylated with calf intestine alkaline phosphatase (Agilent Technologies, Santa Clara, CA, USA), denatured with dimethyl sulfoxide, and labeled with pCp-Cy3 using T4 RNA ligase (Agilent Technologies, Santa Clara, CA, USA). The labeled RNAs were hybridized to custom 8 × 60 K human miRNA microarrays (Agilent Technologies, Santa Clara, CA, USA). Following hybridization and washing, the arrays were scanned with an Agilent microarray scanner using high dynamic range settings as specified by the manufacturer (Agilent Technologies, Santa Clara, CA, USA). Microarray results were extracted using Agilent Feature Extraction software (v12.0; Agilent Technologies, Santa Clara, CA, USA). The total gene signal was normalized to the 75th percentile of the signal intensity.

### 4.11. Target Predictions and Functional Annotation

Gene targets for differentially expressed miRNAs were initially computationally predicted using established miRWalk target-prediction software Version 2.0 [126]. Provided gene targets were uploaded to the online Database for Annotation, Visualization, and Integrated Discovery (DAVID 6.8) for functional annotation and clustering analysis. Genes based on their associated gene ontology annotations, and the related terms, were clustered into groups with enrichment scores calculated from their EASE Score and the modified Fisher exact *p* value [127]. The web server hosts a continuously updated version of the Kyoto Encyclopedia of Genes and Genomes (KEGG) database release 82.1, which provided a relevant search module based on KEGG pathway descriptions. Molecular targets for each miRNA were retrieved and the validated miRNA–target interaction network was obtained from the CyTargetLinker plug-in in the Cytoscape environment 3.9.1 [128]. The network containing interactions between differentially expressed (DE) DE-miRNA and putative targets was constructed and visualized using Cytoscape [129].

### 4.12. miRNA qRT-PCR

Validation of the top 4 differentially expressed miRNAs by qRT-PCR were conducted on a QuantStudio™ 3 platform (Thermo Fisher Scientific, Skokie, IL, USA). miRNAs were reverse transcribed with looped miRNA-specific reverse transcription (RT) primers (Applied Biosystems, Waltham, MA, USA) using the TaqMan miRNA assays. RT reactions were performed in a volume of 15 μL (10 ng of enriched miRNA), on a GeneAmpPCR System 7500 (Applied Biosystems) in the following conditions: 16 °C for 30 min, 42 °C for 30 min, 85 °C for 5 min, and 4 °C on hold. TaqMan assays were run in triplicate using TaqMan Universal PCR Master Mix II without UNG (Applied Biosystems). qRT-PCR cycling conditions were 95 °C for 10 min, followed by 50 cycles of 95 °C for 15 s and 60 °C for 1 min. The qRT-PCR results were normalized (internal control, U6 RNA (RNU6)) and expressed as fold changes. The Ct values were averaged, and the difference between the average RNU6 and the gene of interest Ct (Ct-diff) was calculated using the 2^−ΔΔCT^ method [130].

### 4.13. Multi-Omics and Multivariate Analyses

MixOmics R package [131] was used to perform multivariate analysis for biological datasets, including 5 samples from different 5 types of omics: clinical (nFeature = 10), lipids (nFeature = 312), microRNAs (nFeature = 81), mRNAs (nFeature = 2499) and proteomics (nFeature = 190) data. Through the Multi-block discriminant analysis with DIABLO approach, it integrates different datasets simultaneously, so that the relationships between heterogeneous omics datasets can be investigated. Firstly, we selected the significant features with *p*-value under 0.05 and the value of log_2_ fold change greater than 1 except for the clinical features. After that, the significant features from multi-omics data were input into the Stacked Partial Least-Squares Discriminant Analysis (SPLSDA). The first four components were analyzed, and a circus plot was generated to exhibit significant features in different data types on a circle. The links between the omics represent the strong positive or negative correlations with the correlation value set to r = 0.8. Additionally, a Circos plot was generated with the circlize R package [132] with the correlation matrix calculated by SPLSDA to highlight the strong correlation between only miRNAs and mRNAs. For KEGG pathway mapping, miRDB [133] was used for the target prediction with a score greater than 90 for miRNA features, and UniProt [134] was used to find the corresponding protein-encoding genes for proteomic features. Then, the g:GOSt function of g:Profiler [135] was used to find the corresponding KEGG pathways.

### 4.14. Statistical Analysis

All data are expressed as mean ± standard deviation (SD). The two treatment groups, i.e., OSA and OSAT, were compared by Mann–Whitney *U* test or paired Student’s *t* tests. Multiple group comparisons were done by analysis of variance. All data were analyzed with GraphPad Prism software (9.5.0). OPLS-DA (Orthogonal Projections to Latent Structures Discriminant Analysis) was used to reduce the dimension and identify spectral features driving group separation. Data transformation and multivariate analyses, volcano plots and heatmaps, and orthogonal partial least squares discriminant analysis (OPLS-DA) were carried out as previously described with MetaboAnalyst 5.0 [136]. Receiver Operating Characteristic (ROC) curve analysis was performed to predict the diagnostic effectiveness of biomarkers by MetaboAnalyst 5.0 [136]. The area under the ROC curve (AUC) value was utilized to determine the diagnostic effectiveness in discriminating OSA from OSAT samples. Significance was determined by *p*-values < 0.05 and represented as follows: * *p* < 0.05, ** *p* < 0.01, *** *p* < 0.001, unless indicated otherwise.

## Figures and Tables

**Table 1:** Demographic characteristics of OSA and OSAT subjects.

Term	OSA	OSAT
Age	41.11 ± 8.12	42.13 ± 6.0
BMI, kg/m^2^	30.21 ± 0.51	32.12 ± 3.22
AHI, events/hour	70.03 ± 16.08 *	2.71 ± 2.05 **
Triglycerides (mg/dL)	222.14 ± 74. 18	148.15 ± 78.17 **
Total cholesterol (mg/dL)	234.07 ± 20.14	212.28 ± 33.32 *
HDL cholesterol (mg/dL)	47.01 ± 10.06	42.12 ± 7.13 *
LDL cholesterol (mg/dL)	135.29 ± 13.18	151.16 ± 33.36
Glucose (mmol/L)	101.12 ± 11.16	94.09 ± 14.28 *
SysBP	126.17 ± 10.05	125.22 ± 15.15
DyBP	82.11 ± 8.13	73.16 ± 10.05 *
SpO2 during wake (%)	90.50 ± 2.87	94.21 ± 1.07 **

* Indicates *p*-value < 0.01, while ** *p*-value < 0.001.

**Table 2:** List of the total lipids that are differentially expressed between the OSAT and OSA groups.

Items	FC	log_2_ (FC)	*p*-Value	=−LOG_10_ (*p*-Value)
LPC O-16:1	0.06	−4.1391	0.003138	2.5034
TG 8:0_8:0_28:4	0.28	−1.8468	0.015079	1.8216
LDGTS 21:0	0.37	−1.4297	0.008058	2.0938
TG 8:0_12:0_38:9	0.48	−1.0739	0.018855	1.7246
TG 8:0_11:0_38:8	0.49	−1.0182	0.041437	1.3826
AHexCer (O-20:5)16:1;2O/14:0;O	0.51	−0.96081	0.019799	1.7034
PC 9:0_42:6	0.53	−0.91807	0.043307	1.3634
DGGA 27:0_17:1	0.56	−0.8414	0.015464	1.8107
TG 8:0_12:0_38:7	0.56	−0.83895	0.034252	1.4653
TG 8:0_10:0_38:7	0.57	−0.81719	0.015201	1.8181
TG 8:0_9:0_36:4	0.6	−0.74876	0.007062	2.1511
TG 8:0_10:0_38:5	0.61	−0.70639	0.017472	1.7577
TG 8:0_8:0_38:4	0.63	−0.65961	0.004549	2.342
TG 8:0_8:0_38:5	0.63	−0.6593	0.014651	1.8341
TG 54:4|TG 18:1_18:1_18:2	0.64	−0.6345	0.000793	3.1007
TG 8:0_8:0_38:6	0.64	−0.65175	0.024064	1.6186
TG 8:0_9:0_38:4	0.65	−0.61458	0.000224	3.6501
PS 41:4	0.66	−0.59339	0.009325	2.0303
DGGA 17:0_27:0	0.66	−0.60012	0.024245	1.6154
TG 8:0_12:0_38:6	0.67	−0.57631	0.039363	1.4049
SM 32:6;2O	0.7	−0.51904	0.010348	1.9851
TG 8:0_8:0_36:3	0.72	−0.47121	0.045847	1.3387
TG 8:0_9:0_28:2	1.41	0.49281	0.034692	1.4598
PC 84:7	1.44	0.52454	0.029241	1.534
TG 17:1_18:1_18:2	1.44	0.52957	0.043477	1.3617
TG 15:0_15:0_17:2	1.47	0.55505	0.032567	1.4872
SL 21:0;O/26:2;O	1.47	0.55466	0.049341	1.3068
SL 21:0;O/26:0;O	1.48	0.56277	0.047757	1.321
TG 47:2|TG 14:0_15:0_18:2	1.49	0.57647	0.029857	1.525
TG 51:0|TG 16:0_17:0_18:0	1.51	0.59701	0.032805	1.4841
TG 48:2|TG 14:0_16:0_18:2	1.53	0.61758	0.046616	1.3315
TG 46:3|TG 10:0_17:1_19:2	1.54	0.62159	0.032146	1.4929
TG O-16:1_18:0_18:0	1.54	0.62365	0.036821	1.4339
TG 45:1|TG 12:0_15:0_18:1	1.56	0.64287	0.030752	1.5121
TG 10:0_18:2_18:2	1.57	0.65003	0.037827	1.4222
TG 49:0|TG 16:0_16:0_17:0	1.58	0.65533	0.006496	2.1874
TG 13:0_13:0_18:2	1.59	0.6695	0.040482	1.3927
TG 46:2|TG 12:0_16:0_18:2.1	1.59	0.66815	0.041077	1.3864
TG 46:2|TG 12:0_16:0_18:2	1.6	0.67826	0.035556	1.4491
TG 42:0|TG 12:0_14:0_16:0	1.6	0.67606	0.035651	1.4479
TG 42:1|TG 10:0_16:0_16:1	1.6	0.67972	0.044905	1.3477
TG 8:0_9:0_26:1	1.61	0.68498	0.01263	1.8986
TG 8:0_8:0_26:2	1.65	0.7221	0.020791	1.6821
TG 48:0|TG 14:0_16:0_18:0	1.65	0.72383	0.039058	1.4083
TG 8:0_9:0_28:1	1.66	0.72772	0.031111	1.5071
TG 48:0|TG 16:0_16:0_16:0	1.66	0.73223	0.035803	1.4461
TG 42:2|TG 8:0_16:0_18:2	1.67	0.7407	0.017513	1.7566
SM 39:2;3O	1.67	0.7361	0.042669	1.3699
TG 8:0_9:0_28:3	1.69	0.75753	0.002915	2.5354
TG 44:0|TG 12:0_14:0_18:0	1.69	0.75964	0.047626	1.3222
TG 12:0_14:0_18:1	1.69	0.75294	0.04945	1.3058
TG 46:1|TG 12:0_16:0_18:1	1.69	0.7595	0.049784	1.3029
SL 21:1;O/26:2;O	1.7	0.76579	0.045348	1.3434
TG 8:0_9:0_30:3	1.72	0.77893	0.033386	1.4764
DGCC 40:0_44:8.1	1.77	0.82628	0.026113	1.5831
TG 8:0_8:0_26:1	1.79	0.84235	0.021518	1.6672
TG 44:2|TG 10:0_16:0_18:2	1.8	0.84756	0.024438	1.6119
TG 43:1|TG 9:0_16:0_18:1	1.8	0.84461	0.037674	1.424
PC 80:2	1.87	0.90004	0.031744	1.4983
DGCC 42:0_44:6	1.94	0.95621	0.034975	1.4562
TG 40:1|TG 8:0_16:0_16:1	1.96	0.97235	0.016498	1.7826
SL 17:0;O/26:2;O	1.98	0.98487	0.004897	2.3101
Cer 9:0;3O/26:2;(2OH)	2.02	1.0143	0.010273	1.9883
DGCC 40:0_44:8	2.05	1.0387	0.014613	1.8353
SL 19:1;O/26:2;O	2.09	1.065	0.005659	2.2473
PS 41:3	2.17	1.1178	0.013763	1.8613
DGCC 40:0_44:7	2.2	1.1372	0.002424	2.6154
DGCC 42:0_44:10	2.24	1.1666	0.010602	1.9746
Cer 9:0;3O/42:0;(2OH)	2.4	1.263	0.001415	2.8492
DG 34:2	2.52	1.3321	0.002642	2.5781
DGCC 38:0_44:5	2.98	1.5758	0.008464	2.0724
HexCer 19:3;3O/26:2;(2OH)	4.06	2.0217	0.006126	2.2128

## Data Availability

All data presented in the study are available upon request from the corresponding author.

## Supplementary Materials

The supporting information can be downloaded at: https://www.mdpi.com/article/10.3390/ijms242216074/s1.
