# Supplementary material for: Multi-Omics Analysis of Circulating Exosomes in Adherent Long-Term Treated OSA Patients
Source: Int J Mol Sci. 2023 Nov 8;24(22):16074. doi: 10.3390/ijms242216074 (PMC10671639; doi:10.3390/ijms242216074)
Supplement: Supplementary file 1 [file ijms-24-16074-s001.zip › ijms-2596792-supplementary.pdf]

Supplementary Figures

Figure S1: Subjects enrollment characteristics who diagnosed with OSA (OSA) and treated with CPAP for 1 year.

Figure S2. Characterization of circulating plasma exosomes derived from OSA and treated for 1 year (OSAT). Transmission electron microscopy (TEM), Nanoparticle Tracking Analysis (NTA) analyzers, Flow Cytometry used to measure plasma exosome size distribution and concentration from OSA and OSAT samples. (a) shows representative EM image of isolated exosomes illustrating the appropriate size (30-120 nm). (b) Nanoparticle tracking analysis (NTA) show the size distribution of paired OSA exosomes. (c) Flow cytometry determination analysis of purified plasma exosomes following specific isolation with magnetic beads stained with anti-CD63, and CD81 shows the presence and absence of exosomes. n=12 Significance was tested by 2-tailed non-parametric Mann-Whitney T test, $p<0.001$ (***).

Figure S3: Confocal microscope images illustrating exosome uptake internalization by naïve human endothelial cells. Exosomes were isolated from plasma of OSA and OSAT subjects and labelled with PKH67. Human endothelial cells were grown on coverslips for 24 h and the labelled exosomes derived from OSA and OSAT with PKH67 were added to the cells at 37°C for 24 h. Exosome uptake was measured using confocal laser scanning microscopy at 490 nm excitation and 502 nm emission. Cells were washed and nuclei (blue) stained with DAPI. As controls, no exosomes were used, but PKH67 was added. n=6. Scale bar: 10 μm.

Figure S4: Lipidomic analysis of OSA exosomes using LC-MS/MS. (A) Supervised OPLS-DA of OSA and OSAT subjects for the differentially expressed lipids for sphingolipids, (B) glycerolipids, (C) phospholipids, and saccharolipids. The red color indicates up-regulation, and the blue color indicates down-regulation. n=12/group

Figure S5: Lipidomic analysis of OSA exosomes using LC-MS/MS. (a) heatmap clustering for the statistically significant sphingolipids lipids (b) glycerolipids, (c) phospholipids, and (d) saccharolipids. The red color indicates up-regulation, and the blue color indicates down-regulation.

Figure S6: Lipidomic analysis of OSA exosomes using LC-MS/MS. (a) Volcano plots for the statistically significant sphingolipids lipids based on log2 fold change and -log10 (p-value), (b) glycerolipids, (c) phospholipids, and (d) saccharolipids. The red color indicates up-regulation, and the blue color indicates down-regulation.

Figure S7: Receiver operating curve (ROC) for differentially expressed exosomal proteins derived from OSA and OSAT subjects. (a) ROC for the Differentially expressed proteins, and (b) representative for ROC such as HSP7C, ATPB and IF5AL proteins.

Figure S8: Receiver operating curve (ROC) for differentially expressed exosomal miRNAs derived from OSA and OSAT subjects. The measurement of ROC plot is done by Area under curve (AUC) ROC. The ROC curve is created by plotting the true positive rate (TPR) against the false positive rate (FPR) at various threshold settings.

Figure S9: Variable plots for the OSA and OSAT multi-omics biomarker panels. (a) Plots involving individual components of each block are plotted out over the space represented by the components of each block.

Figure S10: Significant biomarker discriminating between OSA and OSAT over component 1. Significant biomarker loading weights for blocks of clinical, lipids, proteins, and miRNAs discriminating between OSA and OSAT over component 1 (clinical data, a), component 2 (lipids, b),

component 3 (proteins, c), and component 4 (miRNAs, d). Bar plot names correspond to the feature selected on each block. The length of the bar represented the significance (loading weights) of the selected features, while the color is related to the strain in which the specific feature is most abundant.

Figure S11: Significant biomarker discriminating between OSA and OSAT over component 1. Clustered image heat map analysis of the variables (clinical, lipids, proteomics, and miRNAs) to present multi-omics profiles for each sample. blue color indicates OSA, while red color is OSAT.
