# Supplementary figures and images for: Multi-Omics Analysis of Circulating Exosomes in Adherent Long-Term Treated OSA Patients
Source: Int J Mol Sci. 2023 Nov 8;24(22):16074. doi: 10.3390/ijms242216074 (PMC10671639; doi:10.3390/ijms242216074)

## Slide 1
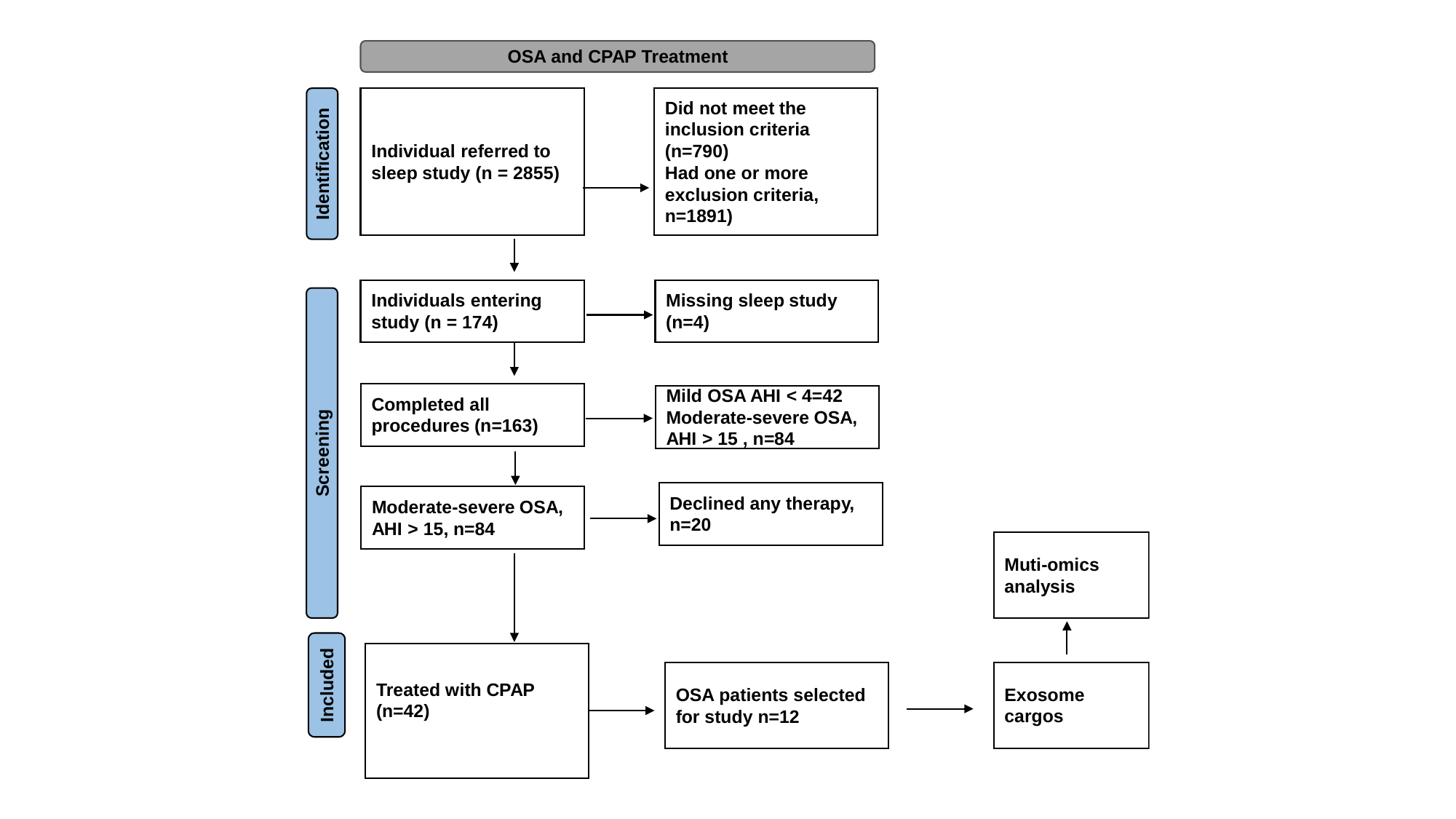

Supplement: Supplementary file 1 [file ijms-24-16074-s001.zip › Figure S1.pptx]

## Slide 1
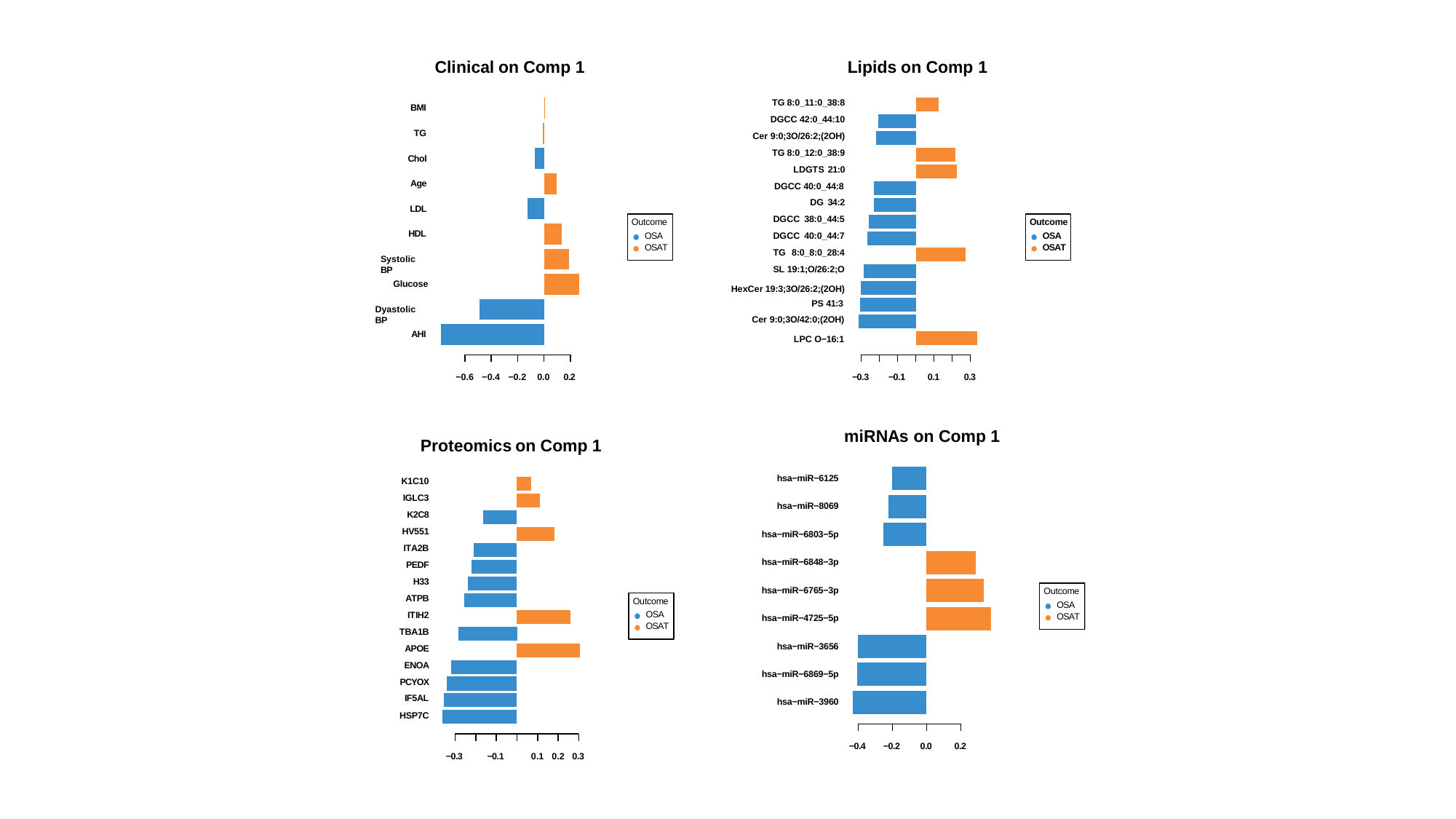

Supplement: Supplementary file 1 [file ijms-24-16074-s001.zip › Figure S10.pptx]

## Slide 1
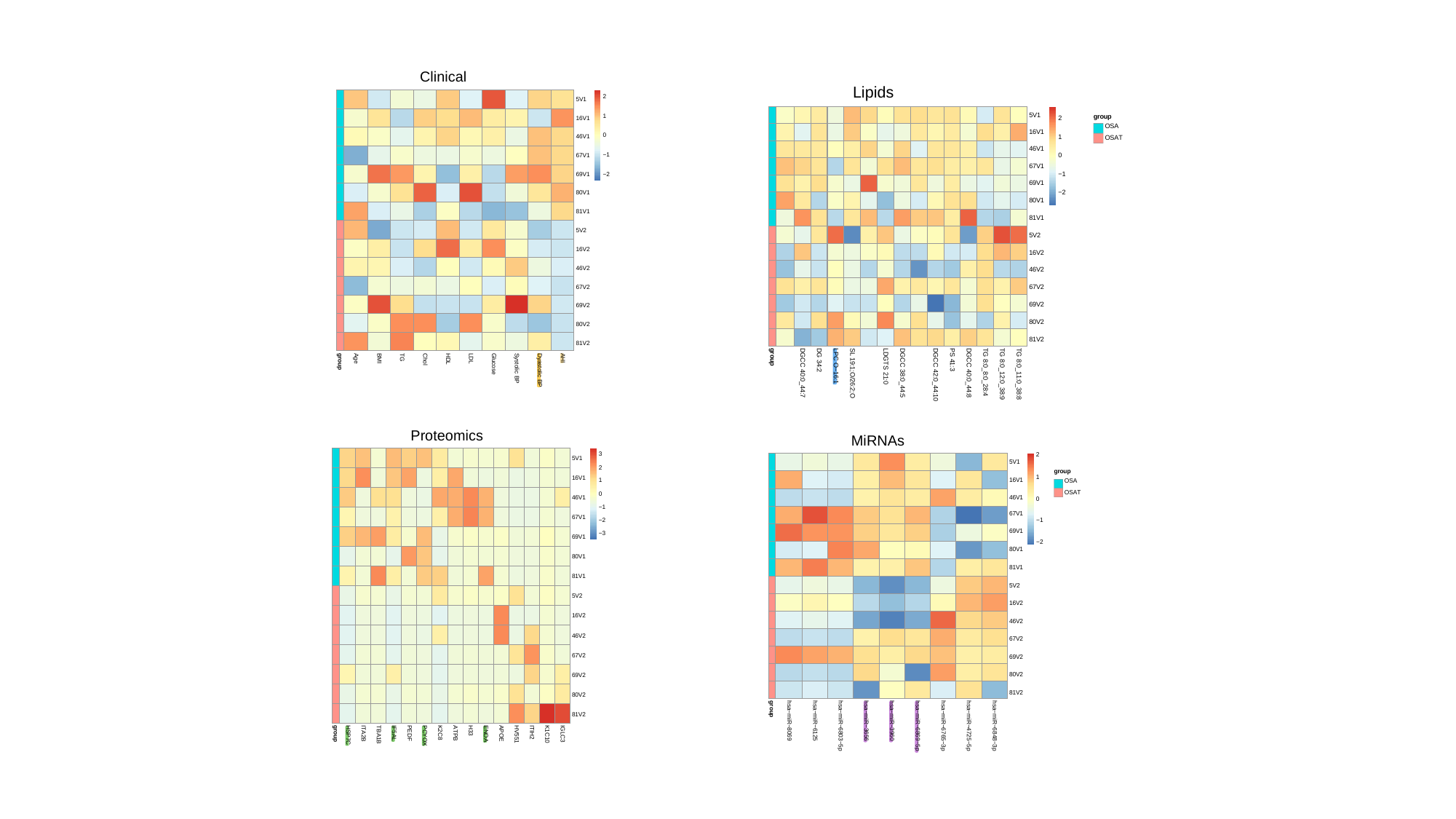

Supplement: Supplementary file 1 [file ijms-24-16074-s001.zip › Figure S11.pptx]

## Slide 1
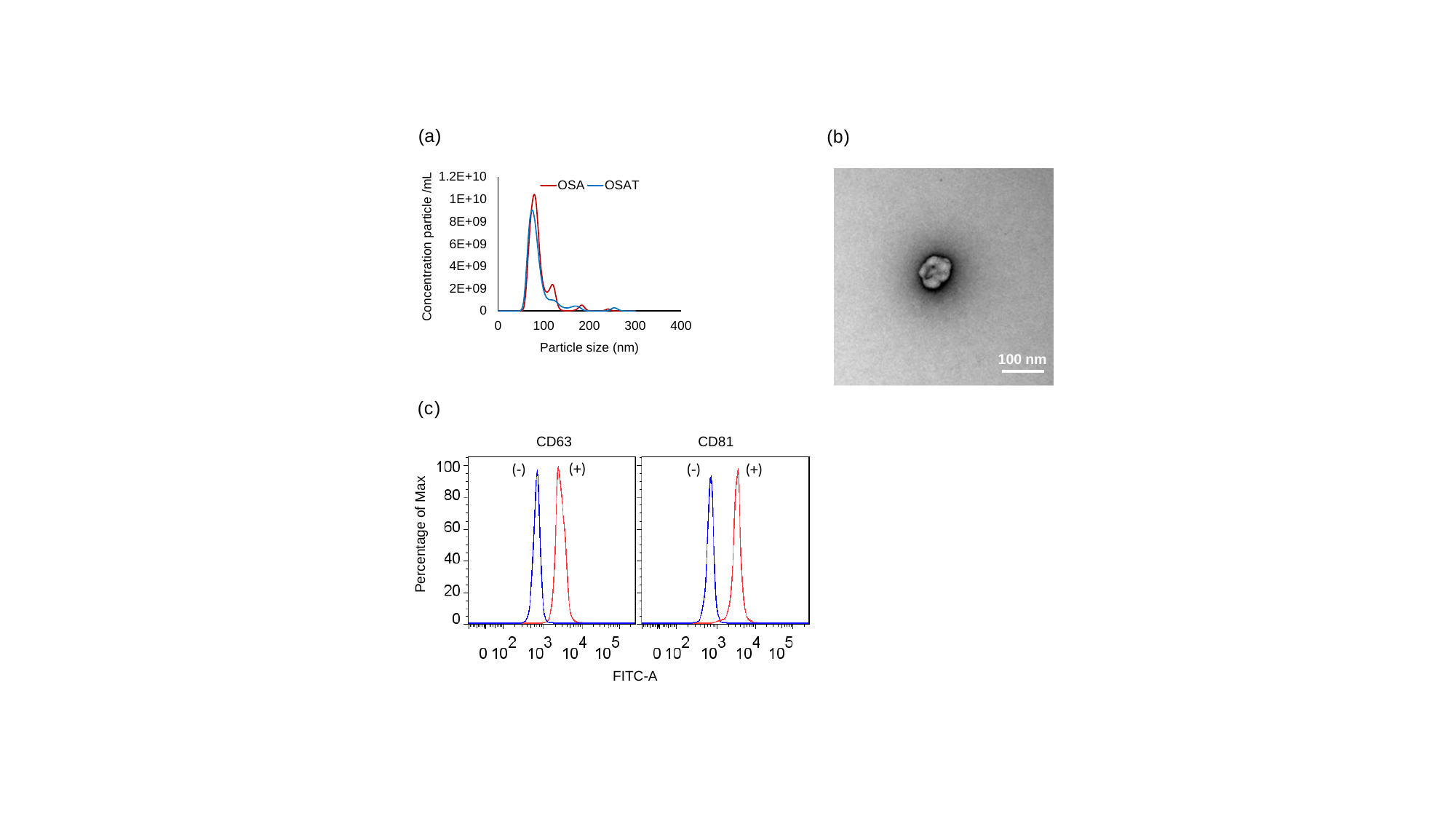

Supplement: Supplementary file 1 [file ijms-24-16074-s001.zip › Figure S2.pptx]

## Slide 1
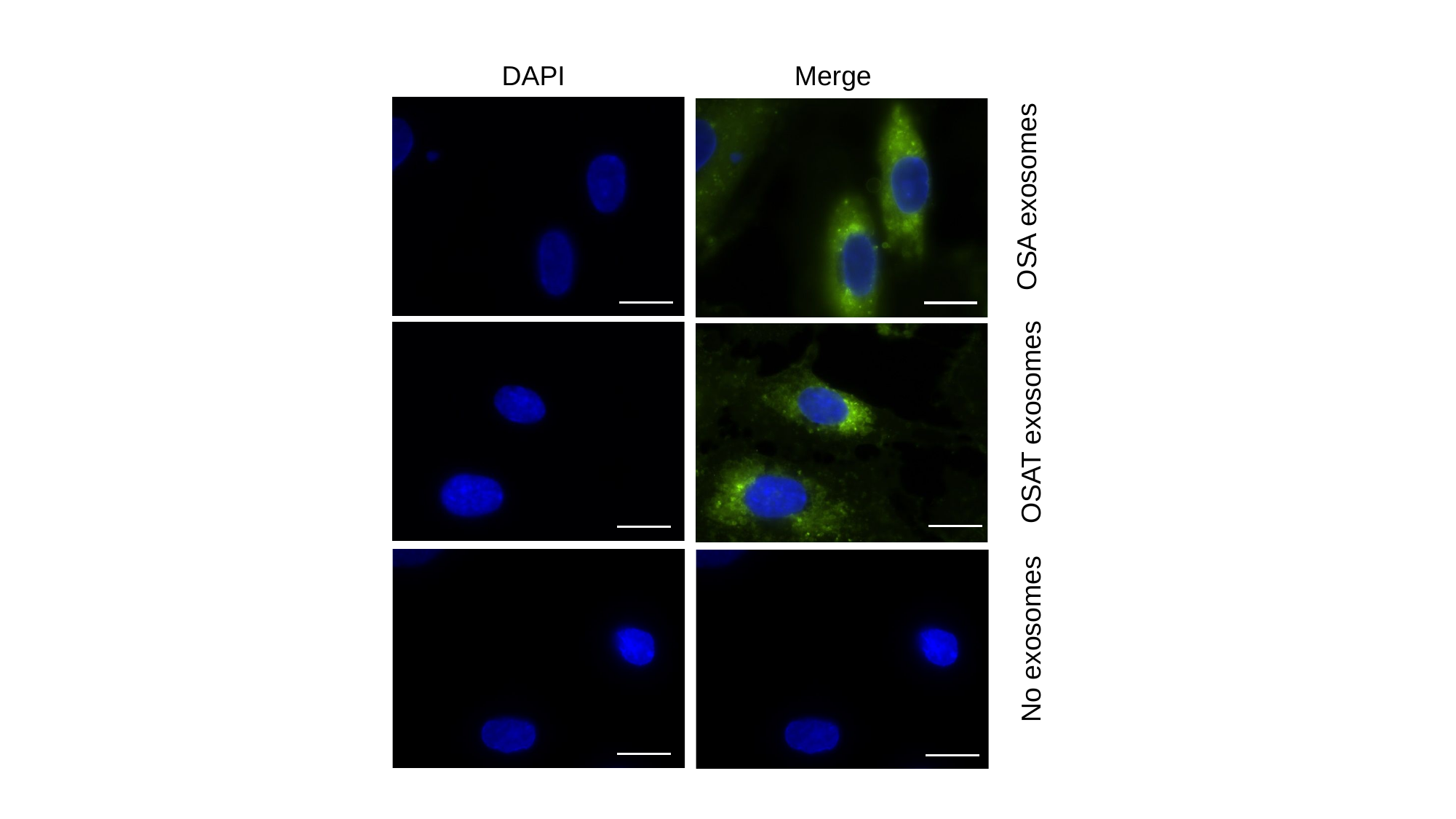

Supplement: Supplementary file 1 [file ijms-24-16074-s001.zip › Figure S3.pptx]

## Slide 1
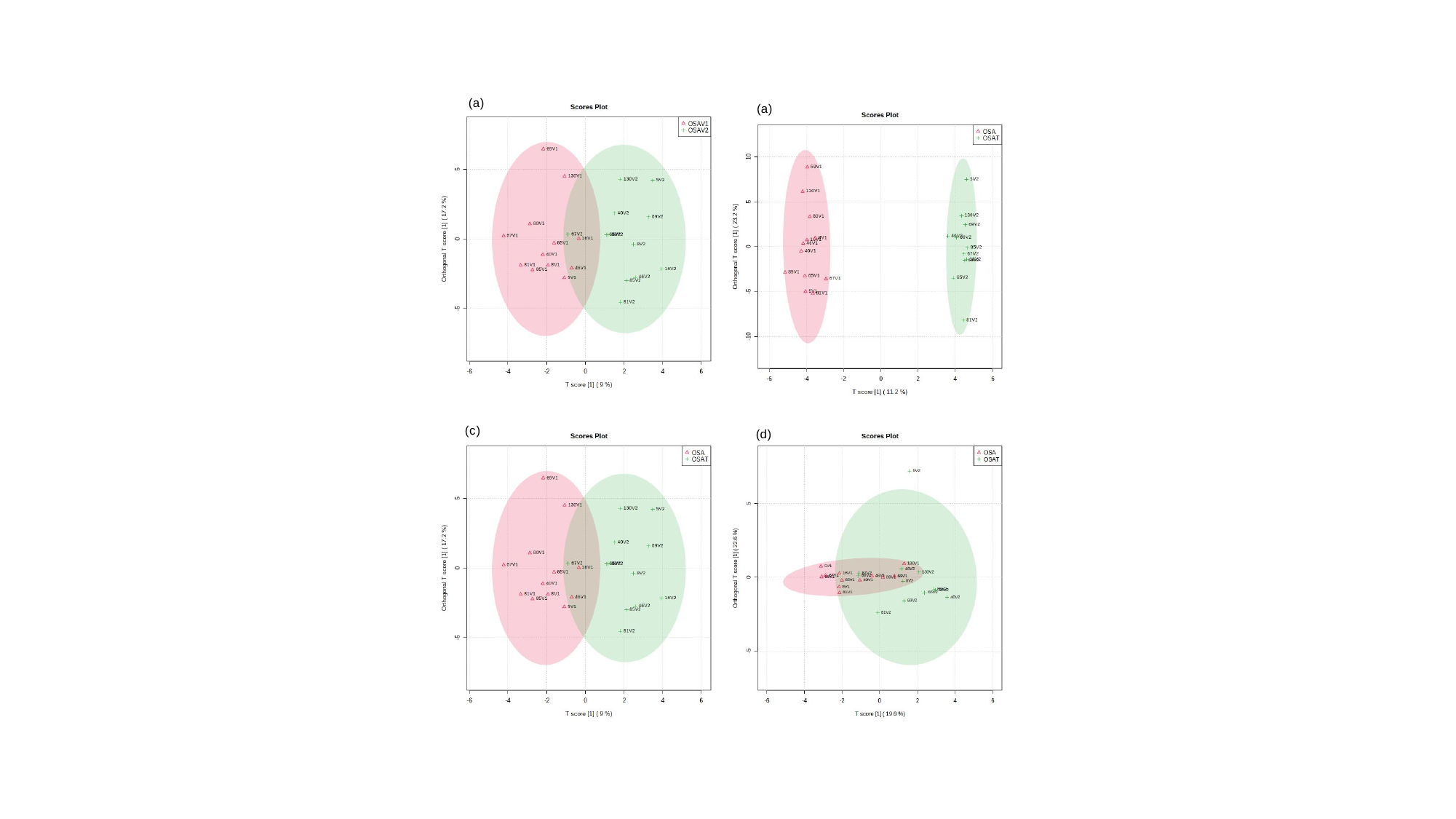

Supplement: Supplementary file 1 [file ijms-24-16074-s001.zip › Figure S4.pptx]

## Slide 1
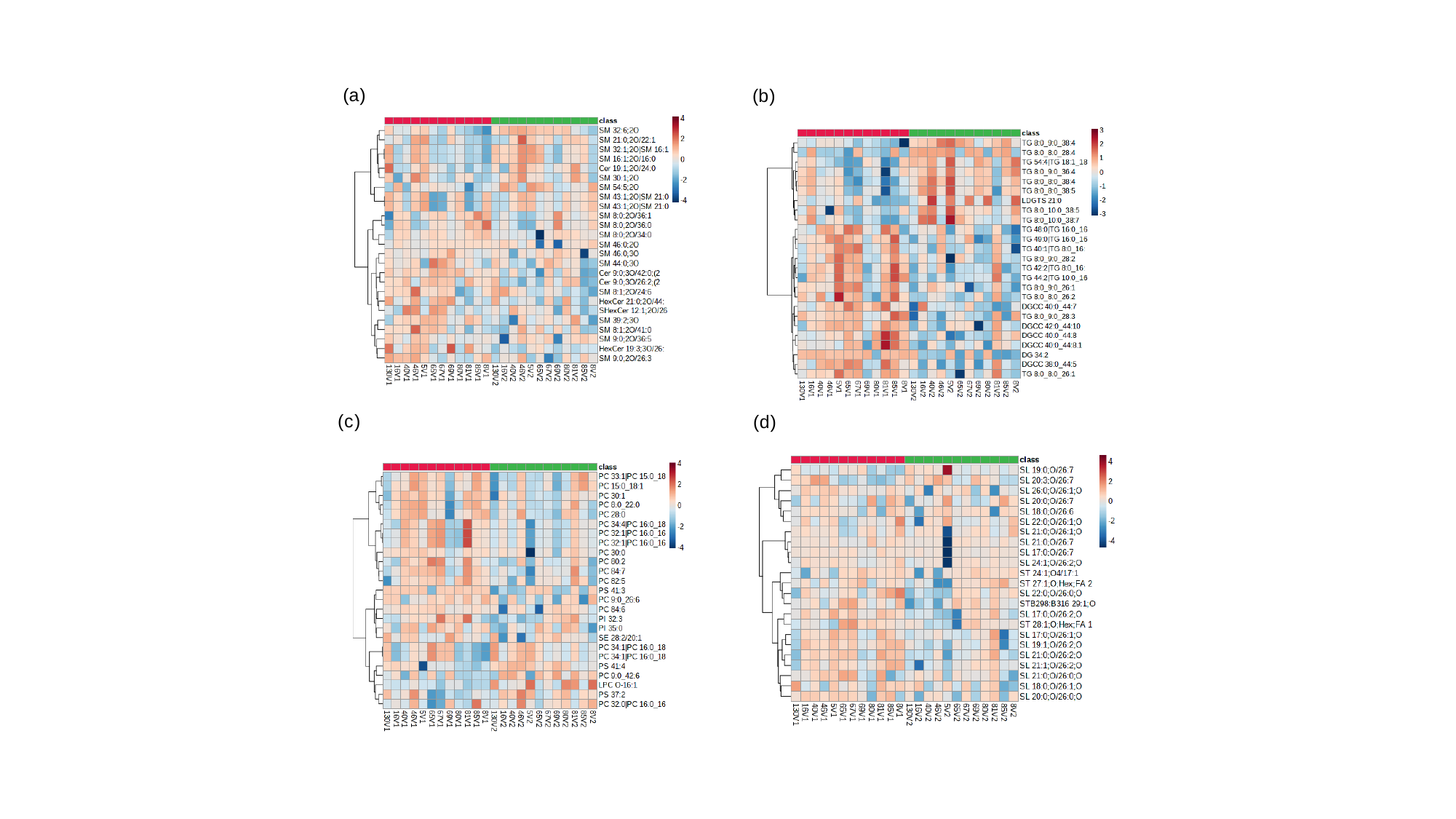

Supplement: Supplementary file 1 [file ijms-24-16074-s001.zip › Figure S5.pptx]

## Slide 1
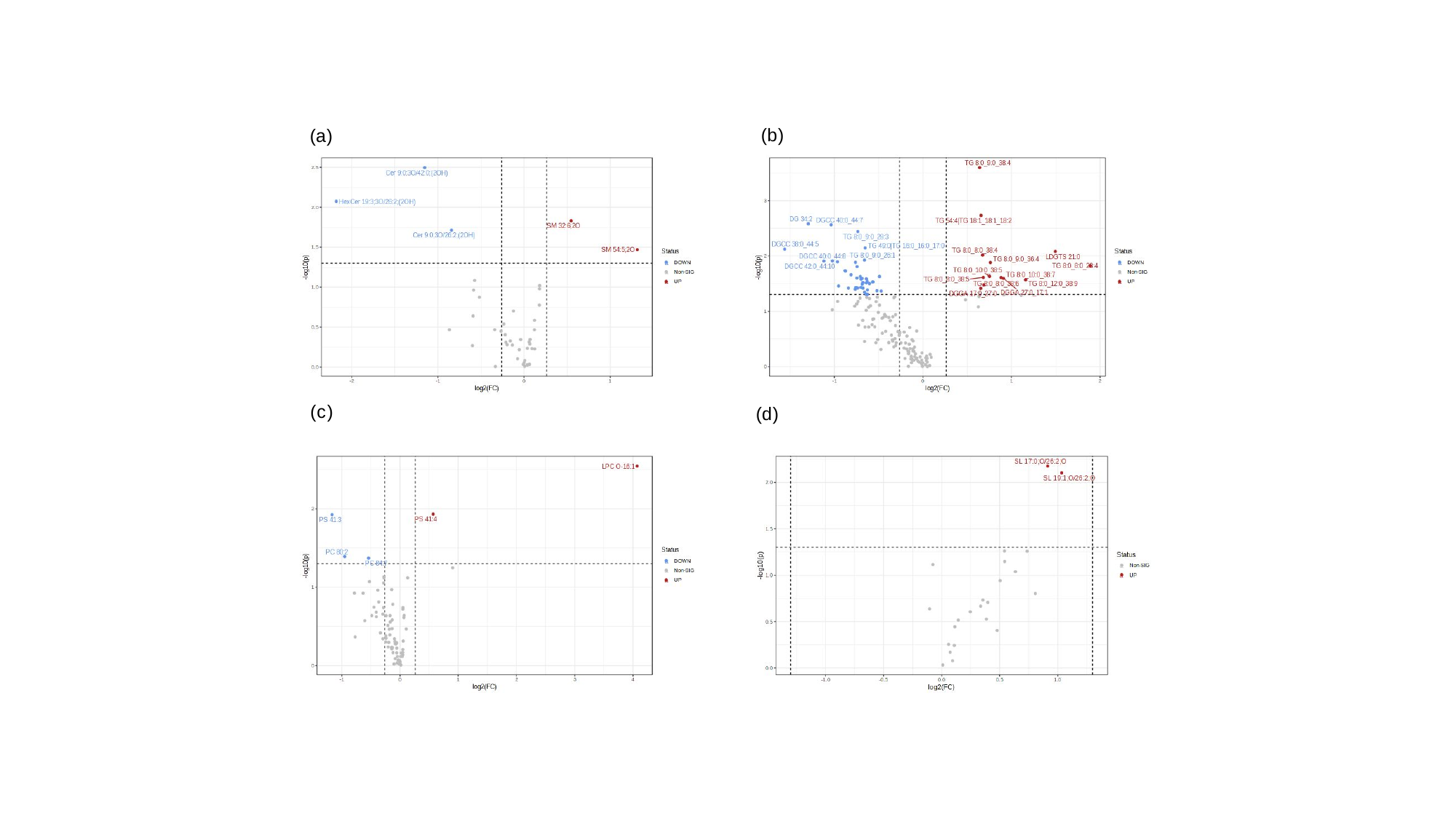

Supplement: Supplementary file 1 [file ijms-24-16074-s001.zip › Figure S6.pptx]

## Slide 1
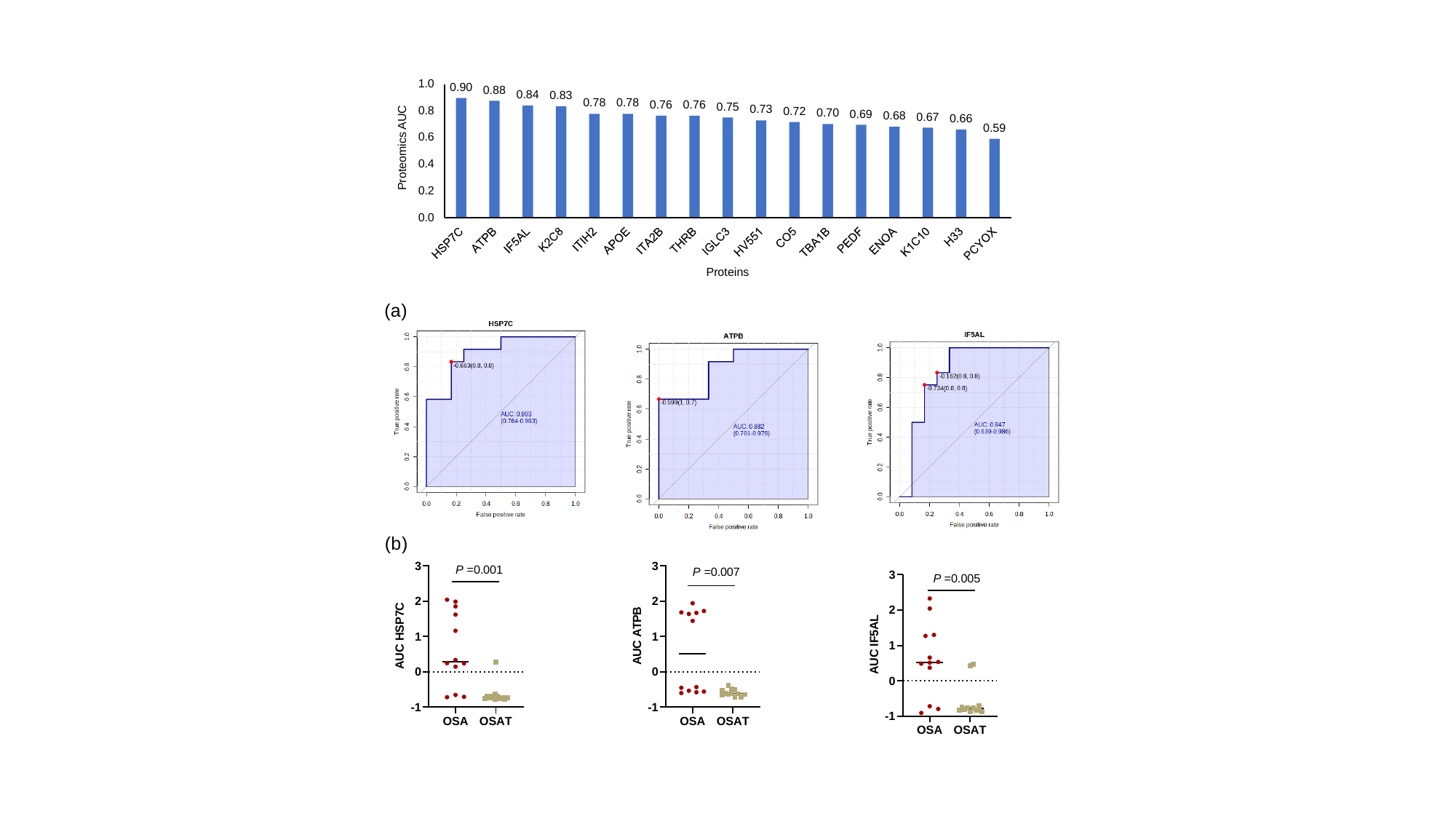

Supplement: Supplementary file 1 [file ijms-24-16074-s001.zip › Figure S7.pptx]

## Slide 1
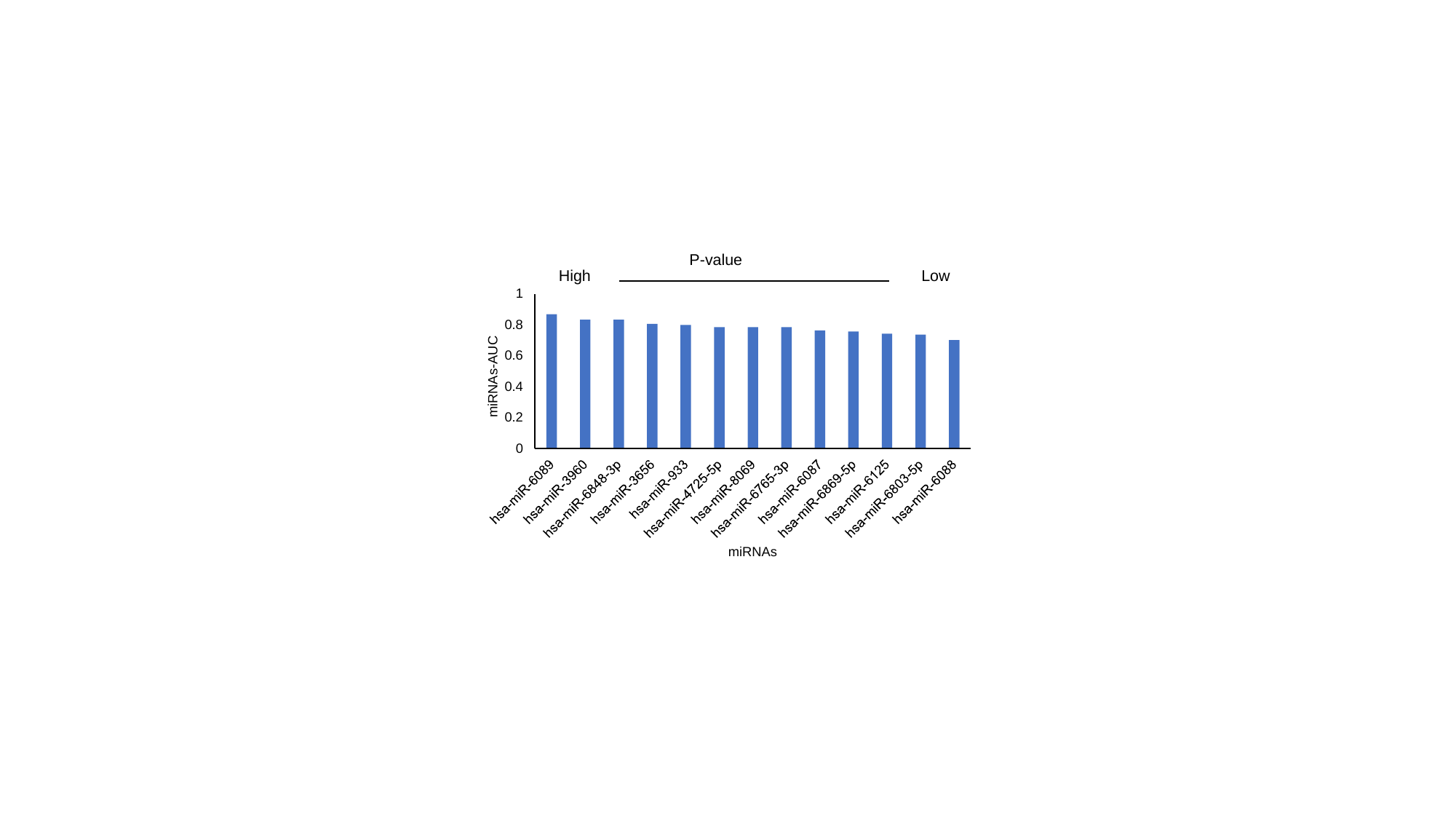

Supplement: Supplementary file 1 [file ijms-24-16074-s001.zip › Figure S8.pptx]

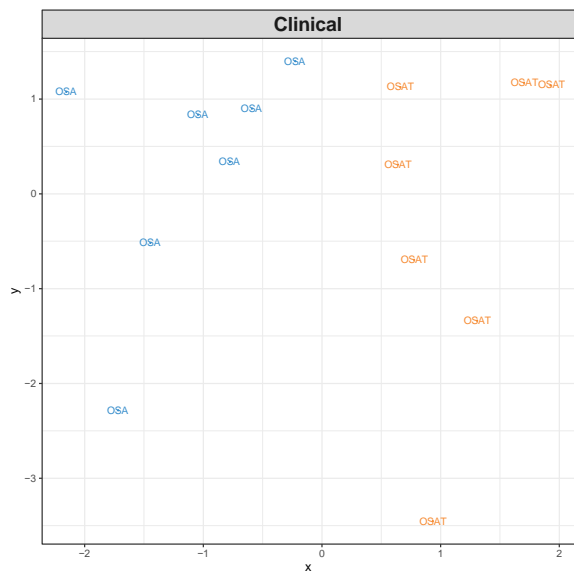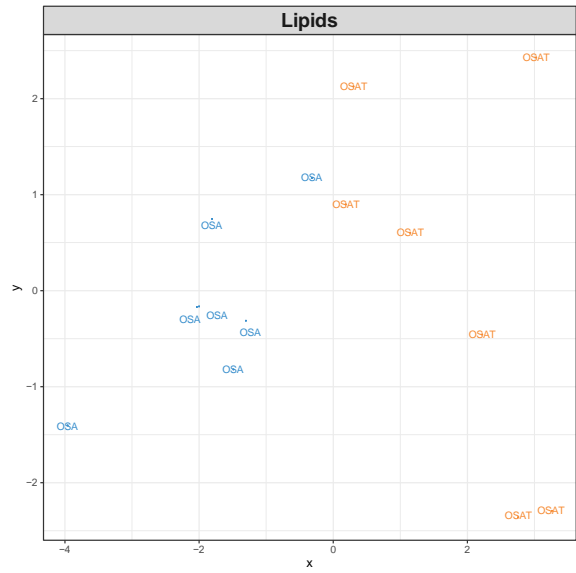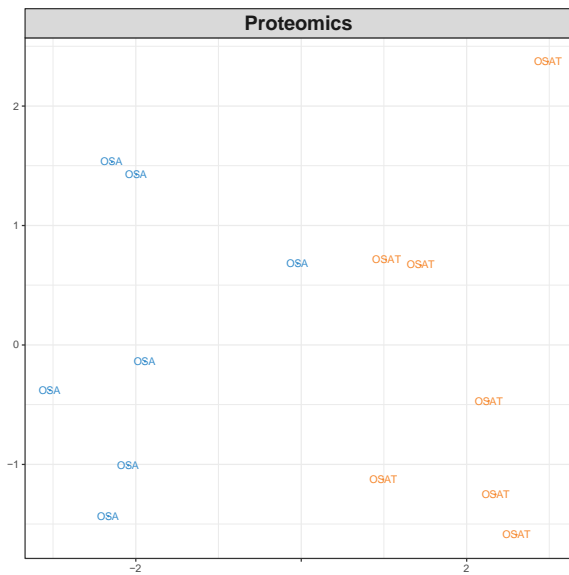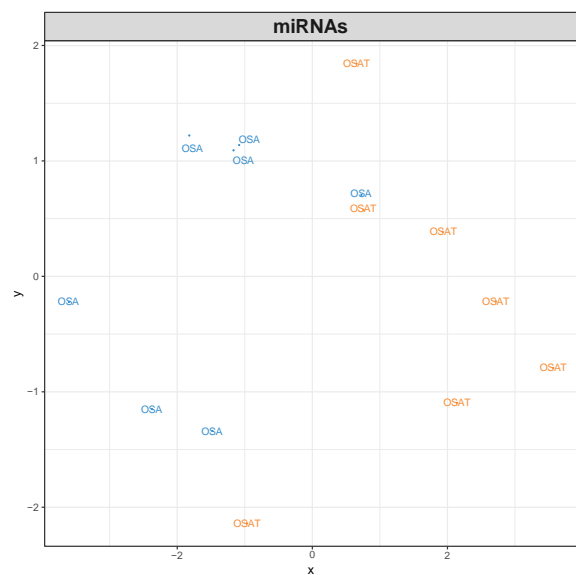

Supplement: Supplementary file 1 [file ijms-24-16074-s001.zip › Figure S9.pdf]
